# Resorbable barrier polymers for flexible bioelectronics

**DOI:** 10.1038/s41467-023-42775-5

**Published:** 2023-11-11

**Authors:** Samantha M. McDonald, Quansan Yang, Yen-Hao Hsu, Shantanu P. Nikam, Ziying Hu, Zilu Wang, Darya Asheghali, Tiffany Yen, Andrey V. Dobrynin, John A. Rogers, Matthew L. Becker

**Affiliations:** 1 Department of Chemistry, Duke University, Durham, NC 27708 USA; 2 Department of Mechanical Engineering, Northwestern University, Evanston, IL 60208 USA; 3 Querrey Simpson Institute for Bioelectronics, Northwestern University, Evanston, IL 60208 USA; 4 Department of Chemistry, University of North Carolina-Chapel Hill, Chapel Hill, NC 27599 USA; 5 Department of Biomedical Engineering and Neurological Surgery, Northwestern University, Evanston, IL 60208 USA; 6 Department of Materials Science and Engineering, Northwestern University, Evanston, IL 60208 USA; 7 Thomas Lord Department of Mechanical Engineering and Materials Science, Duke University, Durham, NC 27708 USA; 8 Department of Biomedical Engineering, Duke University, Durham, NC 27708 USA; 9 Department of Orthopedic Surgery, Duke University, Durham, NC 27708 USA

**Keywords:** Biomedical materials, Polymer synthesis, Polymers

## Abstract

Resorbable, implantable bioelectronic devices are emerging as powerful tools to reliably monitor critical physiological parameters in real time over extended periods. While degradable magnesium-based electronics have pioneered this effort, relatively short functional lifetimes have slowed clinical translation. Barrier films that are both flexible and resorbable over predictable timelines would enable tunability in device lifetime and expand the viability of these devices. Herein, we present a library of stereocontrolled succinate-based copolyesters which leverage copolymer composition and processing method to afford tunability over thermomechanical, crystalline, and barrier properties. One copolymer composition within this library has extended the functional lifetime of transient bioelectronic prototypes over existing systems by several weeks–representing a considerable step towards translational devices.

## Abstract (web summary)

Resorbable bioelectronic devices have potential as tools for monitoring physiological parameters, but short functional lifetimes have slowed translation. Here, the authors report succinate-based copolyesters with barrier properties able to extend the functional lifetime of devices.

## Introduction

**Fig. 1:**
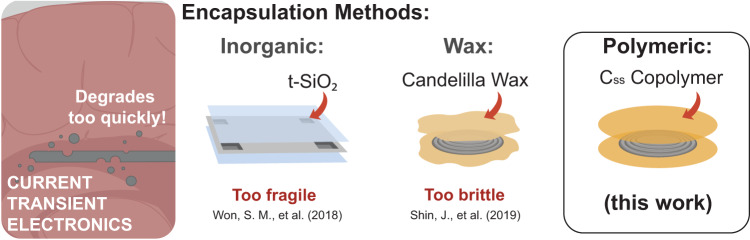
Encapsulation methods for transient bioelectronics. Degradable Mg-based transient bioelectronics have been developed to improve the measurement of intracranial pressure (ICP). Several encapsulation approaches have been developed to increase their functional lifetime in vivo including inorganic coatings, wax, and degradable polymers.

Resorbable, implantable bioelectronic devices have shown promise in a number of pre-clinical applications for continuous monitoring of physiological signals while eliminating the need for secondary removal surgeries^1–10^. At the forefront of these efforts are Mg-based electronics which oxidize readily and dissolve rapidly when placed in contact with bodily fluids^4,5,8,9,11^. However, this rapid degradation results in short functional lifetimes which necessitate the development of barrier technologies to extend their use in vivo. Several approaches, including inorganic substrates, natural wax, and polymer films encapsulation strategies have been developed to this effect (Fig. 1)^5–7,12^. Assessment of these systems has focused on mimicking in vivo device environments by measuring fractional changes in the resistance of encapsulated Mg traces submerged in phosphate-buffered saline (PBS) over time. Wax encapsulations have exhibited the best performance to date under these conditions for homogenous encapsulants; however, the device lifetime (~9−11 days) remain too short to be deployed clinically or commercially^5^. Similarly, inorganic substrates and wax systems are brittle and possess little control over degradation which present additional limitations that are unfavorable for implanted bioelectronic applications^6^.

Synthetic polymer properties have been tuned widely to alter elasticity, flexibility, and degradation^13,14^. Polyanhydrides, gelatin, poly(lactic-*co*-glycolic acid), polyimines, poly(glycerol sebacate), poly(octamethylene maleate (anhydride) citrate), polylactic acid (PLA), and poly(l-lactide-*co*-ε-caprolactone) polymers have each been investigated for barrier film applications^4,7,11,12,15,16^. Indeed, the biggest advancement in functional lifetime to date (~30 days for 300 µm thick films) employed poly(l-lactide-*co*-ε-caprolactone) in micropatterned composites^12^. Barrier properties improved considerably through the strategic inclusion of nanoparticles; however, the pristine polymer only lasted 1.5 days. Similarly, other efforts have focused on physical changes to the encapsulation (e.g., increasing film thickness) or adjusting the device components (less water-sensitive electronics) to reduce device sensitivity and degradability^7^. This leaves a gap in degradable materials which show intrinsically high barrier properties that could be further augmented through additives and other treatments.

The correlation between crystallinity and improved water barrier properties due to increased diffusion path tortuosity is often leveraged to tune polymer systems^17,18^. However, increases in crystallinity can be associated with a reduction in the polymer  flexibility or elasticity necessary to respond to the physical interactions between the device and the tissue environment^18^. Some studies indicate that the type of crystallinity and amorphous phase conformation may be more important than the amount of crystallinity which provides a potential solution to this trade-off^18^. Degradable polymer systems exhibit additional obstacles as barrier encapsulants for transient electronics as swelling during degradation can lead to device delamination and microdefect formation-contributing to device failure long before the barrier films have fully degraded^5,19^.

Barrier materials used for transient device encapsulations must be hydrophobic enough to avoid issues associated with swelling, yet sensitive to physical changes in the device environment in vivo. While hydrophobic materials are available, they are rarely resorbable, and often untested. Predictable degradation of the barrier system is also critical to retaining the benefits of transient devices (e.g., eliminates secondary removal surgeries). Thus, endogenous human metabolites and metabolic byproducts are attractive monomer feedstock due to their inherent biocompatibility and known pathways for degradation products^20–23^. Our previous efforts have demonstrated the utility of a degradable dithiol derivative of succinic acid (C_ss_), a Krebs cycle intermediate, for resorbable polymers^22^.

**Fig. 2:**
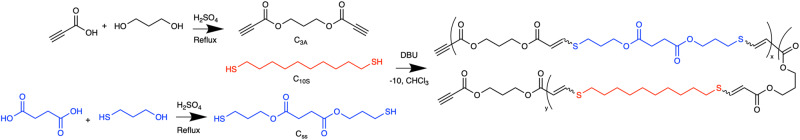
Preparation of stereocontrolled C_SS_ polyester copolymers. C_3A_ and C_SS_ monomer precursor synthesis were carried out via Fischer esterification. After purification, a series of thiol-yne polymerizations were carried out with different stoichiometric ratios of the degradable C_SS_ monomer and 1,10-decanedithiol (C_10S_) to vary the composition of the resulting copolymers. H_2_SO_4_ sulfuric acid, DBU 1,8-Diazabicyclo[5.4.0]undec-7-ene, CHCl_3_ HPLC-grade chloroform.

This work extends our copolyester design by leveraging the metabolizable succinate monomer (C_ss_ in Fig. 2) along with other hydrolytically-degradable ester linkages in the polymer backbone. Paired with stoichiometric variations of a long, hydrophobic dithiol (1,10-decanedithiol), the polymer degradation, mechanical properties, and crystallinity of this copolymer system can each be tuned to maximize the barrier properties. Crystallinity was further maximized through control of the stereochemistry of the alkene in the copolymer backbone imparted by thiol-yne click chemistry. Herein, we report a series of copolymers incorporating degradable C_ss_ monomers which considerably outperform existing bioelectronic water barrier systems by several weeks.

## Results and discussion

### Polymer synthesis and characterization

The copolyester library for the degradable barrier films was synthesized via a stereocontrolled thiol-yne polymerization^22,24–27^. These polymerizations feature well-defined *cis*: *trans* ratios controlled by the nature of the organobase catalyst, solvent polarity (which influences the K_d_ between the organobase and the propriolate monomer), and the reaction temperature. Previous work has shown an empirical correlation between higher *cis* content and increased crystallinity^22^. Therefore, to obtain relatively high (~80%) *cis* stereochemistry, the reaction temperatures are maintained near −10 °C (via a sodium chloride ice bath) with catalytic 1,8-diazabicyclo(5.4.0)undec-7-ene (DBU) in chloroform as outlined in Fig. 2.

The alkyne (C_3A_) and the succinate-based (C_SS_) monomers were synthesized via Fisher esterification reactions and purified via column chromatography. The commercially available 1,10-decanedithiol (C_10S_), was introduced to increase the hydrophobicity of the resulting copolyester polymers. Each of the three monomers and DBU were distilled prior to polymerization. Before quenching the catalyst with BHT, a slight excess of C_3A_ was introduced at the end of the polymerization to cap terminating thiol moieties which serves to avoid post-polymerization reactions. The resulting polymers were precipitated directly from the reaction mixture in diethyl ether yielding polymers of moderate molecular mass (55–95 kDa) with molar mass distributions (*Đ*_M_) near 2 as expected for step-growth polymerizations (Table 1). Copolymer structures were confirmed by ^1^H NMR spectroscopy in supplementary information. Timepoints taken during a 20% C_SS_ polymerization suggest that there is a slight increase in succinate monomer incorporation towards the beginning of the reaction due to decanedithiol’s tendency to freeze (Supplementary Fig. 36). However, due to the nature of typical step-growth reactions, the resulting copolymers remain statistical in nature.

**Table 1:** Summary of the thermomechanical properties of the C_SS_ copolymer library

%C_SS_	*M*_*w*_ (kDa)	*Đ*_M_	*T*_g_ (°C)	*T*_m_ ( °C)	*T*_c_ (°C)	*T*_d, 5%_ (°C)	*E*_0_ (MPa)	*ε*_break_ (%)	UTS (MPa)
0	72.8	2.2	−7	112	61	351	65.8 ± 0.6	1457 ± 112	32.2 ± 3.9
10	81.3	2.5	−10	102	54	354	105.2 ± 1.4	1105 ± 67	26 ± 0.1
15	83	2.7	−10	98	40	357	93.1 ± 2.1	1384 ± 108	25 ± 0.5
20	68.6	2.1	−7	99	30	348	57.2 ± 4.4	1700 ± 31	40.5 ± 2.2
25	81.0	2.3	−9	93	26	359	67.2 ± 1.7	1506 ± 37	25 ± 3.4
30	60.4	2.1	−7	89	19	349	40.9 ± 3.5	1638 ± 33	28.3 ± 1.2
40	57.0	2.1	−7	83	13	349	36.2 ± 2.0	2110 ± 50	36.2 ± 4.1
50	94.9	2.1	−10	74	22	348	36.2 ± 2.0	1943 ± 155	37.4 ± 4.5
60	71.7	2.5	−12	-	-	342	12.0 ± 1.4	2077 ± 78	20.5 ± 0.6
100	86.8	1.4	−12	-	-	358	4.6 ± 0.3	1886 ± 147	8.4 ± 1.9

%C_SS_ % incorporation of the C_SS_ monomer, *M*_w_ weight average molecular mass, *Đ*_M_ molar mass distribution, *T*_g_ Glass transition temperature, *T*_m_ melting temperature, *T*_c_ crystallization temperature, *T*_d_ degradation temperature, ^*a*^*E*_0_ Young’s modulus, ^*a*^ε_break_ elongation at break, ^a^*UTS* ultimate tensile strength. ^*a*^Determined from three or more replicates.

Thermomechanical characterization of these copolymers revealed that melting temperature (T_m_) increased with decreasing C_SS_ stoichiometry. At high C_SS_ stoichiometry (60−100 mol% C_SS_) the absence of a *T*_m_ or *T*_c_ is likely due to the ester moieties disrupting polymer packing to the extent that the material is no longer semi-crystalline. Young’s moduli (*E*_0_) and ultimate tensile strengths (UTS) generally decreased with increasing C_SS_ stoichiometry accompanied by a general increase in elongation at break (*ε*_break_). This is expected given the trends observed in crystallinity. Stoichiometry didn’t appear to have a strong effect on the degradation temperature (*T*_d_) which exceeded 340 °C across all the copolymer compositions. Whereas, in vitro hydrolytic degradation studies trended with C_SS_ stoichiometry indicating slow, tunable degradation profiles (Supplementary Fig. 25) which is favorable for longer-term implants.

**Fig. 3:**
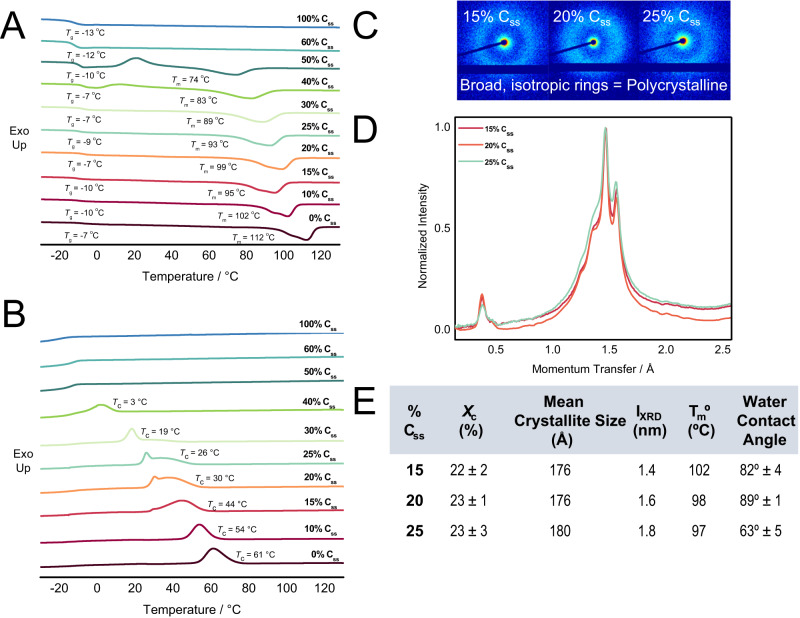
Crystalline properties of C_ss_ copolymers. **A** DSC heating cycle thermograms for the library of C_ss_ copolymer compositions showing that melt transition (*T*_m_) roughly trends with the stoichiometry of the succinate monomer (C_ss_). **B** DSC cooling cycle thermograms for the C_ss_ copolymer library showing that crystallization temperature (*T*_c_) also trends with copolymer composition. **C** 2D Small Angle X-Ray Scattering (SAXS) patterns for 15–25% C_SS_ copolymer stoichiometries. These patterns are consistent with polycrystalline materials and were used to determine the mean crystallite size of each material by averaging across all azimuths. **D** Wide-angle X-ray Scattering (WAXS) of slow-cooled 15–25% C_ss_ showing similar levels of crystallinity and crystalline structure between the three copolymers. **E** Compiled crystalline properties of 15–25% C_ss_ copolymers showing similarity in crystalline properties between these copolymer compositions: *X*_c_ = % crystallinity, I = lamellar thickness determined by the Scherrer equation, *T*_m_° = equilibrium melting temperature determined by extrapolation from variable cooling DSC experiments (Supplementary Fig. 29).

A series of differential scanning calorimetry (DSC) experiments with constant heating and cooling rate (10 °C/min) were conducted to assess the influence of C_SS_ stoichiometry on the thermal transitions (Fig. 3A, B). While the glass transition temperatures, *T*_g_, of the series of polymers was nearly constant (~−7 to −12 °C), the melt transition temperatures, *T*_m_, of the polymers decreased proportionally from 0% C_SS_ (112 °C) to 50% C_ss_ (74 °C). The crystalline behavior and the presence of a *T*_m_ was not observable beyond 50% C_SS_ incorporation after the first heating cycle. The crystallization temperature, *T*_c_, also decreased proportionally from 0% C_SS_ (61 °C) to 40% C_SS_ (13 °C).

The existing correlation between crystallinity and barrier properties necessitated deeper investigation into the crystalline properties of the C_SS_ copolymers (15–25% C_SS_)^18,28^. The 15–25% C_SS_ stoichiometries exhibit similar wide-angle x-ray scattering (WAXS) diffraction patterns which is indicative of similar crystalline structures (Fig. 3C). Degree of crystallinity (*X*_c_) and mean crystallite size were approximately the same across the investigated copolymers (15–25% C_SS_) (Fig. 3D, E) and the observed 2D scattering patterns in SAXS/WAXS are consistent with polycrystalline materials exhibiting many random modes of orientation (Fig. 3E).

**Fig. 4:**
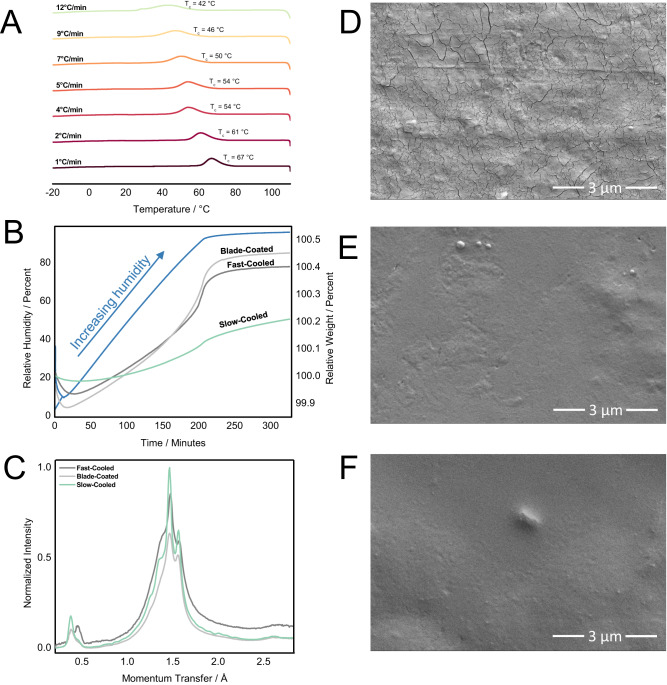
Processing conditions impact surface defects, water penetration, and crystalline properties of C_SS_ copolymer films. **A** Cooling cycle DSC thermogram showing *T*_c_ shifts dependent on cooling rate in the 20% C_SS_ copolymer. This shift in *T*_c_ suggests some difference in the arrangement of the polymer chains as a result of cooling rate. **B** Dynamic vapor sorption (DVS) experiment on a slow-cooled, fast-cooled, and blade-coated 20% C_SS_ copolymer. These experiments suggest that the slow-cooled sample behaves as the best barrier as it absorbed the least amount of water, showed the slowest sorption rate, and the least amount of water absorbed initally. **C** WAXS diffraction pattern of the slow-cooled, fast-cooled, and blade-coated 20% C_SS_ copolymer. The slow-cooled sample showed sharper diffraction peaks of greater intensity which are indicative of higher crystallinity. The slow-cooling and blade-coated methods favored the 16 Å feature whereas the fast-cooling method exhibited two dominant polymorphs. **D** SEM image of compression-molded 20% C_SS_ which was cooled quickly on the benchtop exhibiting many surface defects. **E** SEM image of a 20% C_SS_ controlled-cooling sample prepared by DSC which exhibits very few surface defects. F. SEM image of a blade-coated 20% C_SS_ film which also showed a reduction in surface defects as compared to the compression-molded sample. All SEM images were taken in at least duplicate.

The properties of semi-crystalline polymers are strongly affected by their processing conditions^29,30^. Thus, DSC experiments with varied cooling rates (1–12 °C/min) were conducted to observe how cooling rate after processing may affect polymer properties. These cooling rate experiments demonstrated that the crystalline properties of these polymers varied dramatically with cooling rate where slower cooling rates gave more unimodal, higher temperature crystallization temperatures, *T*_c_ (Fig. 4A). Across all of the C_SS_ polymer stoichiometries, low *T*_g_s (sub-ambient) are observed which could partially explain the observed dependence in crystallinity on cooling rate as polymer systems demonstrate higher dynamic mobility above the *T*_g_^31^. This was further supported by the observation of very thin lamellar crystals which suggests that these structures are unstable (Fig. 3E)^32,33^. Despite this, the *X*_c_ did not appear to change significantly when the polymer was stored at ambient temperature for several weeks (${\chi }_{c,{fresh}20\%{Css}}=$23.1% vs. ${\chi }_{c,{aged}20\%{Css}}=$23.0%) though the mean crystallite size decreased approximately 14 Å between the freshly pressed and aged, slow-cooled 20% C_SS_ substrates (176 Å and 162 Å respectively).

Scanning electron microscopy (SEM) imaging of 20% C_SS_ films shed light on the differences in film surface quality following several thermal processing methods. The samples prepared by controlled-cooling methods via DSC or which were blade-coated appeared more uniform with fewer observable defects in comparison to the fast-cooled compression-molded samples (Fig. 4D–F). This could be due to the observed dependence in crystallinity on cooling rate or differences in shrinkage^34^. The slow-cooled sample also demonstrated low surface roughness as determined by atomic force microscopy (RMS roughness = 9.7 ± 3.1 nm for slow-cooled 20% C_SS_). Low surface roughness is important for consistent barrier performance as non-smooth surfaces can exhibit thinner regions which fail before the rest of the film.

WAXS diffraction patterns of 20% C_SS_ also showed that the processing method affected the homogeneity and size of the observed features between 13.6–16.6 Å (Fig. 3B). The larger feature predominated, and the extent of crystallinity (${\chi }_{c}$) was greater following longer cooling times (${\chi }_{c,{fastcooling}}=$ 15.6% vs. ${\chi }_{c,{slowcooling}}=$ 23.1%) or when blade-coated (Fig. 4C). Comparison of the first DSC heating thermogram for fast and slow-cooled films also suggests one favored polymorph for the slow-cooled sample as opposed to two for the fast-cooled film (Supplementary Fig. 34). Variable temperature WAXS (VT-WAXS) experiments showed crystal growth in both slow (stepped) and fast-cooling conditions that resulted in a difference in the size and number of crystalline features (Supplementary Fig. 33). The cooling rate appears to affect the short-range order of amorphous compositions like the 60% C_SS_ copolymer as seen in both VT-WAXS and variable cooling rate DSC experiments (Supplementary Fig. 27).

### Barrier assessment of C_SS_ copolymers

Examination of C_SS_ crystallinity provides insight into ideal processing methods which maximize *X*_c_ and surface uniformity but cannot be used to draw direct conclusions about the barrier properties of the polymers. Dynamic vapor sorption (DVS) experiments with the 20% C_SS_ copolymer processed by three different methods confirmed that processing method considerably affects the rate and amount of water absorption (Fig. 4B). While blade-coating appeared to reduce the surface defects observed by SEM (Fig. 4C), it also showed reduced crystallinity when compared to the thermally processed samples. Overall, the slow-cooled melt-pressed sample showed the greatest crystallinity and barrier effect.

**Fig. 5:**
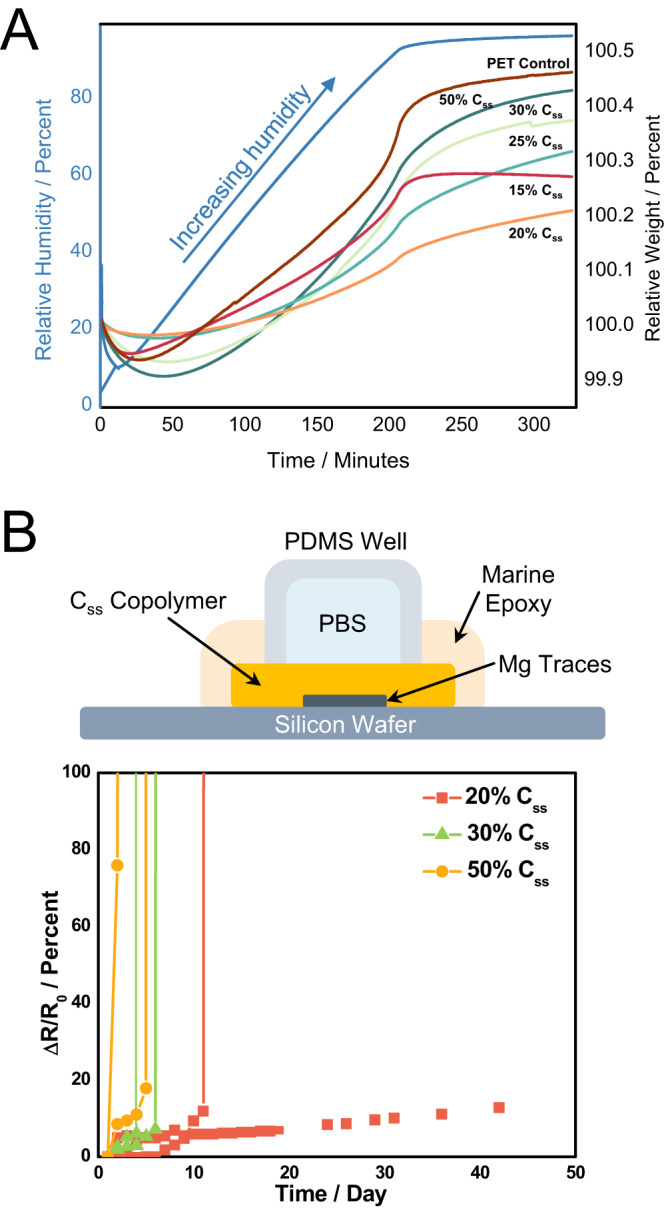
Preliminary water barrier assessment. **A** Comparison of weight change over time between different copolymer compositions over a 0.5%/min range from 0 to 97.5% relative humidity. Water absorption rate appears to increase with increasing succinate stoichiometry and each C_SS_ copolymer absorbed less water than the PET control. 20% C_SS_ appears to absorb the least amount of water of the copolymers investigated. **B** Change in resistance over time of three C_SS_ copolymer compositions encapsulating serpentine magnesium traces in the experimental setup depicted above the plot. These results suggest barrier performance trends with the stoichiometry of the succinate-based monomer and show that the 20% C_SS_ copolymer can be viable to extend the lifetime of these devices over 40 days at 37 °C. This surrogate system exhibited some variability in performance which is likely due to micro/nanodefects during the fabrication process.

DVS experiments with a subset of the synthesized copolymers demonstrated that succinate stoichiometry directly trends with water absorption until the 20% C_SS_ copolymer (Fig. 5A). Notably, each of the tested copolymers demonstrated slower rates of water absorption (shallower slope) than poly(ethylene terephthalate) (PET) thin films.

A subset of C_SS_ copolymer was used to encapsulate serpentine magnesium traces at 37 °C against the perpendicular diffusion of PBS in the experimental setup shown in Fig. 5B meant to mimic Mg-based transient systems. The performance of these copolymers shows a similar trend in barrier performance to that of the DVS experiments, where barrier ability appears to increase with decreasing succinate stoichiometry. The 20% C_SS_ copolymer was able to extend the lifetime of these model devices over 40 days–making this composition a promising candidate for translational Mg-based implantable sensors. While the measurements are not directly on the implanted devices and controlling the thermal properties during the device fabrication will certainly need further optimization, the change in resistance over time is significantly less than other copolymer stoichiometries and the timelines are approaching clinical and commercial relevance. This surrogate system also exhibited some variability in performance which is likely due to micro/nano defect formation in the tested films.

### Molecular dynamics simulations

**Fig. 6:**
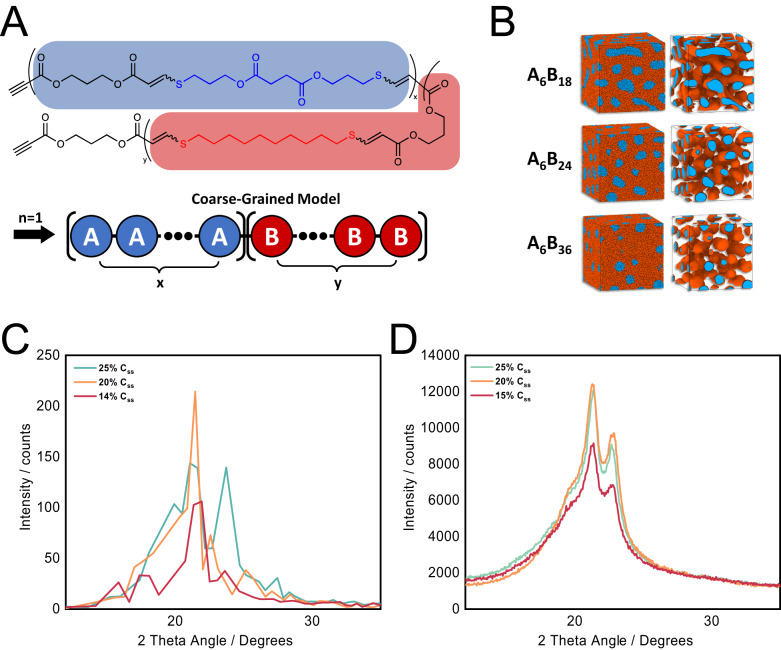
Molecular dynamics simulations of this copolymer system. **A** Coarse-graining scheme of a copolymer chain, where monomer pairs C_3A_–C_SS_ and C_3A_–C_10S_ are modeled as beads of type A (blue) and type B (red) respectively. **B** Morphology of self-assembled structures changes from spheres to intertwined cylinders (gyroid-like structure) of A domains. A_6_B_18_ = 25% C_ss_, A_6_B_24_ = 20% C_ss_, A_6_B_36_ = 14% C_ss_. Scattering function S(q) for three self-assembled structures in **B**. **D** X-ray diffraction of 10–25% C_SS_ similar levels of crystallinity and crystalline structure between the three copolymers. This data also agrees with the scattering functions in **C**.

Molecular dynamics simulations of a subset of C_SS_ stoichiometries provided insight into why the 20% C_SS_ copolymer performed best despite exhibiting similar degrees of crystallinity to the neighboring stoichiometries. These simulations suggest that these copolymers self-assemble due to the differences in polarities of C_SS_ and C_10S_ (Fig. 6). To quantify the relative polarity of the chain segments, the partial charge distributions were calculated for three monomer sequences by using B3LYP 6-31 G* (d,p) basis set for Austin Model 1 (AM1) optimized structures implemented in Gaussian 09. The results of this calculations show that C_SS_ has the highest level of polarity (the magnitude of variation of partial charges between groups of atoms) among all three monomer sequences (Supplementary Fig. 31). The alkyl chain in the central segment in C_10S_ is symmetric and appears to be fully screened. Therefore, the order of the polarity for three repeats is C_SS_ > C_3A_ > C_10S_. Considering the regularity of the sequence distribution in a copolymer chain and their relative polarity, two neighboring monomer sequences C_3A_-C_SS_ and C_3A_-C_10S_ pairs were treated as coarse-grained beads of type A (blue, more polar) and B (red, less polar) respectively (Fig. 6A).

To make the system as simple as possible while still obtaining meaningful information, simulations were performed on copolymer chains with *n* = 1 in a melt. The list of the studied systems is summarized in Supplementary Table 2. Figure 6B shows snapshots of the simulation box for copolymers with different composition which corresponding scattering functions are plotted in Fig. 5C. The observed trend of scattering intensity with copolymer composition is consistent with X-ray diffraction data shown in Fig. 6D. Thus, the nonmonotonic dependence of intensity could be explained by transformation of the self-assembled structures from spheres to intertwined cylinders (gyroid-like morphology) of A-blocks. The additional scattering functions and snapshots of the simulation box illustrating morphology transformation with copolymer composition are shown in Supplementary Fig. 32. This difference in assembled structure could provide insight into superior barrier performance exhibited by the 20% C_SS_ copolymer with respect to other stoichiometries.

### Biocompatibility studies

Implantable devices, particularly those which are intended to degrade in vivo, must be verified for tissue compatibility. Disks of a subset of the synthesized copolymers with 200 µm thickness (100 µm thick samples can be found in Supplementary Fig. 38) along with PTFE controls, were implanted subcutaneously for 10 weeks to observe their degradation behavior and tissue inflammatory responses in vivo. PTFE is a suitable control as it is widely used for medical device fabrication, does not degrade over time, shows excellent barrier properties, and provides corrosion resistance^35^.

**Fig. 7:**
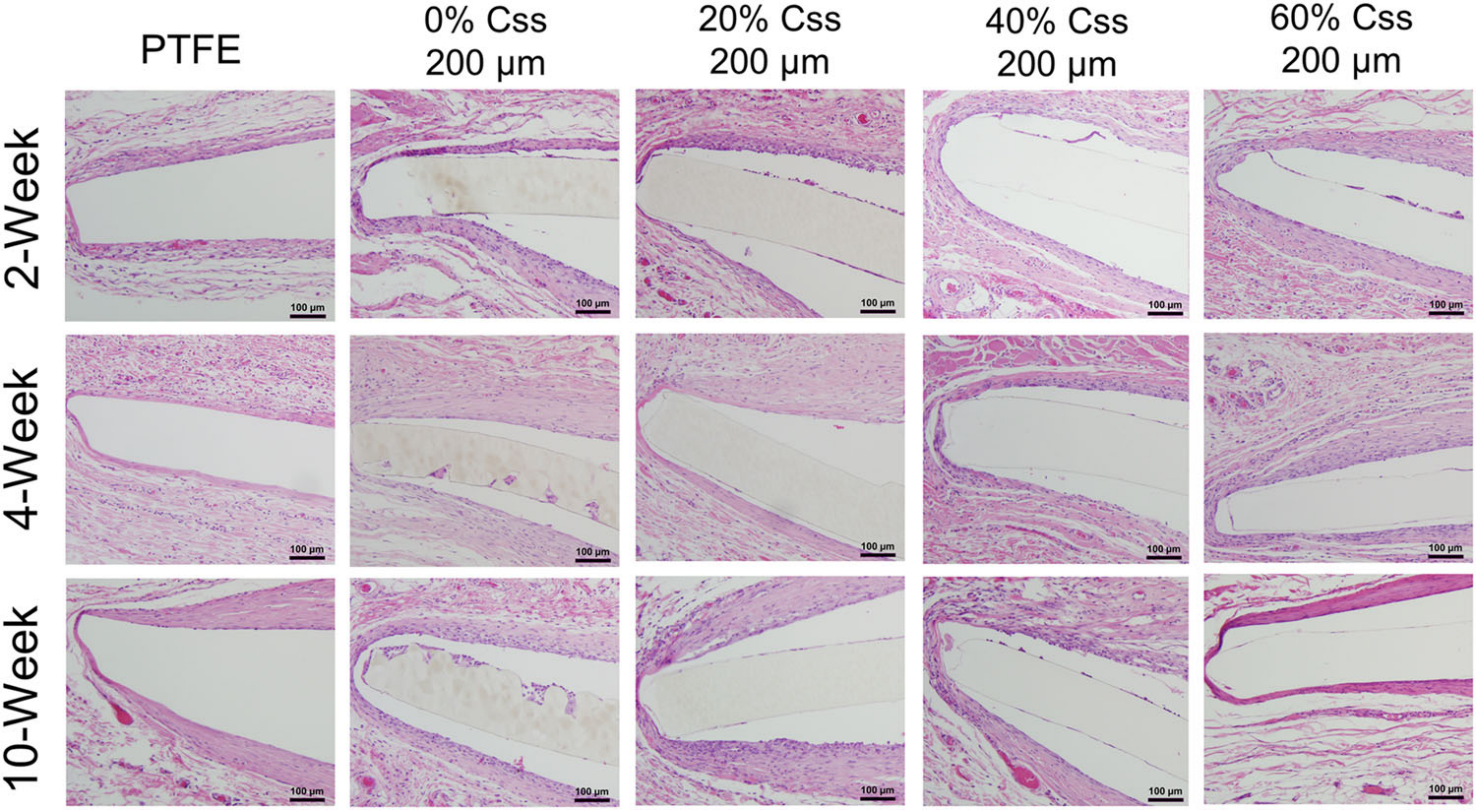
In vivo tissue compatibility studies performed on copolymer samples in a murine subcutaneous implantation model. C_ss_ copolymer disks (200 µm) were implanted subcutaneously for up to 10 weeks to observe their degradation behavior and tissue inflammatory responses in vivo. Hemotoxylin and Eosin staining was used to detect fibrous capsule and presence of inflammatory cells based upon ISO 10993-6 standards as assessor metrics (see Supplementary Table 1). The presence of inflammatory cells diminished over time of implantation in all groups between week-2 and week-10 timepoints. Necrosis was absent in all sample groups and multinucleated giant cells were either sparse or lacking along with no evidence of immunogenicity. Comparisons to PTFE controls indicated that good biocompatibility and biostability (*n* = 8).

No gross inflammation was evident from macroscopic observations postmortem for any of the polymers. Across all C_SS_ copolymer groups, fibrous capsule formation was found to be <100 μm and uncalcified, which indicated that no severe inflammatory response occurred towards the copolymer and is within the range of an accepted response for an implantable material (Fig. 7). Supplementary Table 1 shows that the presence of inflammatory cells diminished over time in all groups between 2- and 10-week timepoints. The absence of lymphoid cell aggregation or cuffing of the surrounding blood vessels suggested no immunogenicity. Furthermore, there was no evidence of necrosis noted and multinucleated giant cells were either sparse or lacking in the capsule walls. Ultimately, comparison of the tissue-material interfacial region between the C_SS_ copolymers and PTFE controls indicated the implanted polymers were biocompatible and biostable (Fig. 7).

### Device prototyping

**Fig. 8:**
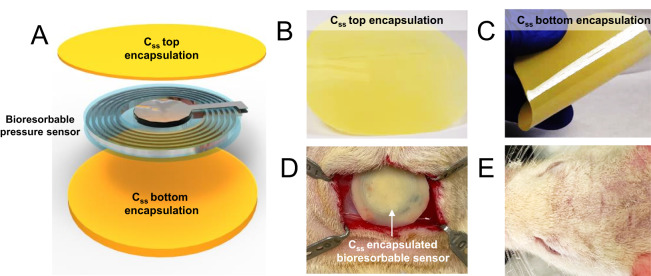
Bioresorbable pressure sensor prototype encapsulated with 20% C_SS_ copolymer films. **A** Schematic illustration showing the bioresorbable pressure sensor sandwiched by two C_ss_ films via melt compression. **B** Photograph of the top of the encapsulation shown in schematic A (25 µm thick). **C** Photograph of the bottom film of the C_SS_ encapsulation (200 µm thick). **D** Photograph of a C_SS_-encapsulated device mounted above an opened craniotomy in a rat model. **E** Photograph showing the incision healing two weeks post-surgery.

In a demonstration of the prototype, a bioresorbable pressure sensor, using the same core materials and structures as described in the previous literature^36^, has been successfully encapsulated with 20% C_SS_ polymers (Fig. 8A). Heat compression for 30 minutes sealed the edges between the top and bottom C_SS_ films (thickness: 25 µm for top and 200 µm for bottom; photographs shown in Fig. 8B, C). Figure 8D presents the device’s implantation into a craniotomy in a rat model. Notably, the incision healed effectively within two weeks post-surgery, as shown in Fig. 8E. It’s worth noting that further improvements are essential in optimizing interlayer adhesion and controlling temperature to enhance crystallinity in the device barrier films.

### Comparison to other systems

**Fig. 9:**
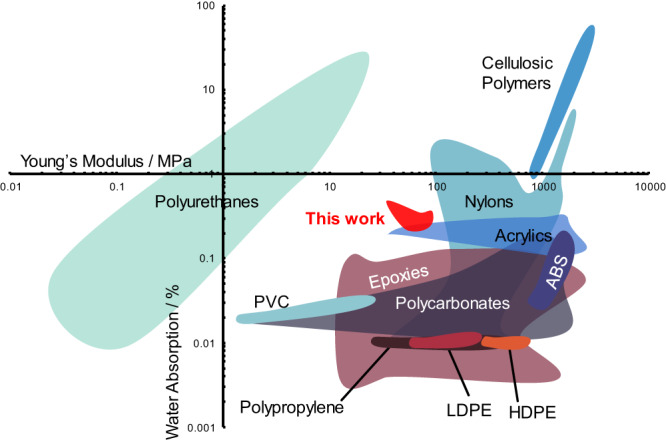
Ashby plot of materials characterized for water absorption and Young’s modulus. This work demonstrates greater flexibility for similar barrier performance to Nylons. Homogenous, nonproprietary polymer systems (not reinforced or filled) were manually compiled from Matweb.com^38^. When presented with a range of values the lower value was used in this plot.

The lack of standardization in assessing barrier performance makes it difficult to draw direct comparisons between systems. However, for systems which measured encapsulation ability of Mg traces this system shows a marked increase in functional lifetime in a thinner film regime than what was previously reported as state-of-the-art (100 µm thick as opposed to 300 µm thick films) without any additives^12^. This system also exhibits a higher Young’s modulus than other systems for this application while still demonstrating sensitivity to the device enviroment^7,16^. Compared to other degradable polymers such as poly(caprolactone) (PCL) or PLA, this system appears to exhibit lower water sorption (20% C_SS_ = 0.2094%, PCL ~ 0.75%^36^, PLA ~ 0.9^37^); however, different characterization methods make it impossible to draw definitive conclusions from this data. This work exhibits greater flexibility for similar water absorption behavior compared to commercially available unreinforced/unfilled polymer systems like nylons (Fig. 9).

### Challenges

This system presents considerable advantages–namely, the promising thin-film barrier performance accompanied by suitable flexibility, biocompatibility and slow degradation. However, the barrier properties of this system should be rigorously investigated to understand how additional factors like density, crystallinity, molecular mass and mass distribution affect observed barrier performance. The current studies present some variability in device performance (Fig. 5B) which we attribute to confounding variables like sealing considerations between the thin-film and device as well as somewhat limited thermal processing capabilities in an academic lab. Validation of these data by DVS experiments provide additional support for our findings, but should be further validated to understand how film dimensions influence these results. Commercial-grade film preparation in a clean room and further testing will be necessary in drawing more direct comparisons between this system and commercial barrier polymers.

This system investigates a narrow monomer scope which balances a metabolizable monomer (C_SS_) with a non-metabolizable monomer (C_10S_). The most promising stoichiometry (20% succinate monomer content) contains relatively low amounts of the metabolizable monomer. Thus, structures which increase the amount of resorbable components while maintaining performance will be an active area of future work towards increasing tunability in degradation time.

### Outlook

The combination of stereochemistry, stoichiometry and optimized thin-film processing has resulted in a series of degradable barrier films that show promising results in flexible and implantable devices. In particular, a 20% C_SS_ stoichiometry has demonstrated its ability to increase the functional lifetime of Mg-devices to more than 40 days, which is significantly longer than the best performing encapsulation films in the literature. With degradability and semi-crystallinity as keystones in designing the C_SS_ copolymers, these materials have exhibited excellent barrier properties for encapsulating transient bioelectronics. Additional work is required to fully understand the structure-property relationships which govern the barrier properties within this system and a broader monomer scope would enable increased tunability of functional lifetime to better meet clinical targets within this application. Application of this current succinate-based thiol-yne polymer system could be expanded to other transient electronic modalities and applied to other barrier-type industry problems (e.g., packaging). Ultimately, this work represents an exciting step towards translational transient systems.

## Methods

### Materials

All reagents and solvents were used as received without further purification except 1,10-decanedithiol which was distilled under vacuum before use. Chloroform-*d* (CDCl_3_) (99.96 atom% D, contains 0.03% (v/v) TMS), amylene-stabilized HPLC-Grade chloroform (CHCl_3_) (≥99%), Ethyl acetate (EtOAc) (≥99.5%), magnesium sulfate (MgSO_4_) (≥99.5%), sodium bicarbonate (NaHCO_3_) (≥99.7%), sulfuric acid (95-98%), 1,8-diazabicyclo[5.4.0]undec-7-ene (DBU) (98%), hexanes (≥98.5%), butylated hydroxytoluene (BHT) (≥99%), succinic acid (≥99%), 1,3-propanediol (98%) were purchased from Sigma-Aldrich. Propiolic acid (98%) and diethyl ether (Et_2_O) (≥99%) were purchased from Fischer Scientific. 3-mercapto-1-propanol(≥97%), 1,10-decanedithiol (≥98%), were purchased from Tokyo Chemical Industry Ltd.

### Synthesis of 1,3-propane diyl dipropiolate (C_3A_)

The synthetic procedure for C_3A_ was reported previously^20^. A 500 mL one-neck round-bottom flask was charged with 1,3-propanediol (20.000 g, 0.263 mol), propiolic acid (50.000 g, 0.714 mol), and two drops of H_2_SO_4_ in ~200 mL of toluene. The mixture was heated to reflux (130 °C) overnight with a Dean-Stark trap precharged with ~8 mL of toluene to collect the water byproduct. The reaction solution was cooled to ambient temperature and neutralized with a saturated solution of sodium bicarbonate until no longer bubbling, then concentrated and washed with sodium bicarbonate two more times. The organic phase was collected, dried over magnesium sulfate, gravity filtered, and reduced in volume to dryness by rotovap. The collected crude product was then purified through silica column chromatography using ethyl acetate: hexanes (1:3, v/v; *R*_f_ = 0.45) as an eluent. The first spot was distilled under vacuum (0.02 Torr, 110 °C) to yield a colorless oil (39.80 g, 84.0%). ^1^H NMR (500 MHz, CDCl_3_) *δ* 4.29 (t, *J* = 10 Hz, 4H), 2.91 (s, 2H), 2.07 (quint, *J* = 10 Hz, 2H). Note that foil was used to facilitate heat-transfer between the round bottom and condenser in both the fischer esterification and distillation.

### Synthesis of bBis(3-mercaptopropyl) succinate (C_ss_)

The synthetic procedure for C_ss_ was reported previously^18^. Succinic acid (15.000 g, 0.127 mol), 3-mercapto-1-propanol (25.000 g, 0.271 mol), and two drops of H_2_SO_4_ were added to a round-bottom flask (250 mL) in ~200 mL of toluene. The mixture was then heated to reflux (130 °C) with a Dean-Stark trap precharged with ~8 mL of toluene overnight to collect the water byproduct. The reaction solution was cooled to ambient temperature and neutralized with a saturated solution of sodium bicarbonate, then extracted into ethyl acetate three times. The organic phase was collected, dried over magnesium sulfate, gravity filtered, and reduced in volume to dryness. The collected crude product was then purified through silica column chromatography using ethyl acetate: hexanes (2:3, v/v; *R*_f_ = 0.45). The final product was further purified by distillation under high vacuum (0.02 Torr, 200 °C) to yield a colorless oil (26.70 g, 79.1%). ^1^H NMR (500 MHz, CDCl_3_) *δ* 4.20 (t, *J* = 10 Hz, 4H), 2.62-2.57 (m, 8 H), 1.93 (quint, *J* = 10 Hz, 4H), 1.39 (t, *J* = 10 Hz, 2 H). Note that para-toluene sulfonic acid (0.05 eq) can be used in lieu of H_2_SO_4_ as an acid catalyst for similar results. Additionally, foil was used to facilitate heat-transfer between the round bottom and condenser in both the fischer esterification and distillation.

### Representative procedure for thiol-yne step-growth polymerization

80% *Cis*, 20% **C**_**ss**_ copolymer was synthesized as follows: **C**_**ss**_ (0.715 g, 0.268 mmol, 0.2 eq), **C**_**3A**_ (2.42 g, 13.4 mmol, 1 eq) and **C**_**10S**_ (2.21 g, 10.7 mmol, 0.8 eq) were added to an oven-dried 100 mL round-bottom flask with 25 mL HPLC-Grade CHCl_3_ (amylene-stabilized). It is important to use amylene-stabilized chloroform as ethanol inhibitors can undergo a hydroxy-yne reaction which is unfavorable in reaching full conversion. The solution was then cooled to −10 °C (ice and sodium chloride mixture) with stirring for 15 min. DBU (20 μL, 0.134 mmol, 0.01 eq) was diluted in a small amount of CHCl_3_ and added dropwise due to the exothermicity of the reaction. Additional CHCl_3_ was added as needed (i.e., following increases in viscosity which rendered the stir bar unable to mix the reaction solution). Addition of DBU was paused if reaction mixture started to bubble vigorously. 5 min after full addition of DBU, the reaction was allowed to warm to room temperature and stirred under ambient conditions for 2 hours. A few drops of C_3A_ were added prior to quenching to cap terminating thiols, then BHT (225 mg, 10.22 × 10^−5 ^mol) was added to quench the catalyst. Polymer was precipitated directly from the reaction mixture with diethyl ether (400 mL) and collected by decanting the supernatant. The polymer appears white to pale-yellow and was rinsed one additional time with diethyl ether before drying under high vacuum at ambient temperature for 24 h (20% **C**_**SS**_, 8.81 g, 90%). SEC (CHCl_3_, based on PS standards) *M*_n_ = 32.7 kDa, *M*_w_ = 68.6 kDa, *Ð*_M_ = 2.1. DSC: *T*_g_ = −7.3 °C, *T*_c_ = 30.3 °C, *T*_m_ = 99.3 °C. TGA: *T*_d_ = 348 °C. Tensile tests: *E*_*0*_ = 57.2 ± 4.4 MPa, *ε*_break_ = 1700 ± 31%, UTS = 40.5 ± 2.2 MPa. Note that these reactions are extremely sensitive to stoichiometric imbalance and will not go to high conversion if monomers are impure or weighed out carelessly (all monomers were weighed out to ±0.0002 g of the calculated mass). Additionally, if monomers are mixed on ice as opposed to mixed before transferring to ice, C_10S_ monomer is prone to freezing and can cause a slight gradient effect (see Supplementary Fig. 36).

0% **C**_**ss**_: The polymer was prepared by the general procedure described above, but with 0 eq of C_ss_. ^1^H NMR (CDCl_3_, 500 MHz)% *cis* ~ 80 with 0% **C**_**ss**_ incorporation. SEC (CHCl_3_) *M*_n_ = 33.1 kDa, *M*_w_ = 72.8 kDa, *Ð*_M_ = 2.2. DSC: *T*_g_ = −6.8 °C, *T*_c_ = 61.4 °C, *T*_m_ = 112.4 °C. TGA: *T*_d_ = 351 °C. Tensile tests: *E*_*0*_ = 65.8 ± 0.6 MPa, *ε*_break_ = 1457 ± 112%, UTS = 32.2 ± 3.9 MPa.

10% **C**_**ss**_: The polymer was prepared by the general procedure described above but with 0.1 eq of C_ss_ and 0.9 eq of C_10S_. ^1^H NMR (CDCl_3_, 500 MHz)% *cis* ~ 80 with 10% **C**_**ss**_ incorporation. SEC (CHCl_3_) *M*_n_ = 32.1 kDa, *M*_w_ = 81.3 kDa, *Ð*_M_ = 2.5. DSC: *T*_g_ = −9.7 °C, *T*_c_ = 54.0 °C, *T*_m_ = 102.3 °C. TGA: *T*_d_ = 354 °C. Tensile tests: *E*_*0*_ = 105.2 ± 1.4 MPa, *ε*_break_ = 1105 ± 67%, UTS = 26 ± 0.1 MPa.

15% **C**_**ss**_: The polymer was prepared by the general procedure described above but with 0.15 eq of C_ss_ and 0.85 eq of C_10S_. ^1^H NMR (CDCl_3_, 500 MHz)% *cis* ~ 80 with 15% **C**_**ss**_ incorporation. SEC (CHCl_3_) *M*_n_ = 23.6 kDa, *M*_w_ = 54.5 kDa, *Ð*_M_ = 2.3. DSC: *T*_g_ = −10.1 °C, *T*_c_ = 44.1 °C, *T*_m_ = 95.4 °C. TGA: *T*_d_ = 357 °C. Tensile tests: *E*_*0*_ = 93.1 ± 2.1 MPa, *ε*_break_ = 1384 ± 108%, UTS = 25 ± 0.5 MPa.

25% **C**_**ss**_: The polymer was prepared by the general procedure described above but with 0.25 eq of C_ss_ and 0.75 eq of C_10S_. ^1^H NMR (CDCl_3_, 500 MHz)% *cis* ~ 80 with 25% **C**_**ss**_ incorporation. SEC (CHCl_3_) *M*_n_ = 34.0 kDa, *M*_w_ = 89.0 kDa, *Ð*_M_ = 2.2. DSC: *T*_g_ = −9.1 °C, *T*_c_ = 26.1 °C, *T*_m_ = 93.1 °C. TGA: *T*_d_ = 359 °C. Tensile tests: *E*_*0*_ = 67.2 ± 1.7 MPa, *ε*_break_ = 1506 ± 37%, UTS = 25 ± 3.4 MPa.

30% **C**_**ss**_: The polymer was prepared by the general procedure described above but with 0.3 eq of C_ss_ and 0.7 eq of C_10S_. ^1^H NMR (CDCl_3_, 500 MHz)% *cis* ~ 80 with 30% **C**_**ss**_ incorporation. SEC (CHCl_3_) *M*_n_ = 29.1 kDa, *M*_w_ = 60.4 kDa, *Ð*_M_ = 2.1. DSC: *T*_g_ = −7.3 °C, *T*_c_ = 18.5 °C, *T*_m_ = 88.9 °C. TGA: *T*_d_ = 349 °C. Tensile tests: *E*_*0*_ = 40.9 ± 3.5 MPa, *ε*_break_ = 1638 ± 33%, UTS = 28.3 ± 1.2 MPa.

40% **C**_**ss**_: The polymer was prepared by the general procedure described above but with 0.4 eq of C_ss_ and 0.6 eq of C_10S_. ^1^H NMR (CDCl_3_, 500 MHz)% *cis* ~ 80 with 40% **C**_**ss**_ incorporation. SEC (CHCl_3_) *M*_n_ = 26.7 kDa, *M*_w_ = 57.0 kDa, *Ð*_M_ = 2.1. DSC: *T*_g_ = −7.4 °C, *T*_c_ = 12.7 °C, *T*_m_ = 82.5 °C. TGA: *T*_d_ = 349 °C. Tensile tests: *E*_*0*_ = 36.2 ± 2.0 MPa, *ε*_break_ = 2110 ± 50%, UTS = 36.2 ± 4.1 MPa.

50% **C**_**ss**_: The polymer was prepared by the general procedure described above but with 0.5 eq of C_ss_ and 0.5 eq of C_10S_. ^1^H NMR (CDCl_3_, 500 MHz)% *cis* ~ 80 with 50% **C**_**ss**_ incorporation. SEC (CHCl_3_) *M*_n_ = 45.4 kDa, *M*_w_ = 94.9 kDa, *Ð*_M_ = 2.1. DSC: *T*_g_ = −9.7 °C, *T*_c_ = 21.5 °C, *T*_m_ = 74.2 °C. TGA: *T*_d_ = 348 °C. Tensile tests: *E*_*0*_ = 36.2 ± 2.0 MPa, *ε*_break_ = 1943 ± 155%, UTS = 37.4 ± 4.5 MPa.

60% **C**_**ss**_: The polymer was prepared by the general procedure described above but with 0.6 eq of C_ss_ and 0.4 eq of C_10S_. ^1^H NMR (CDCl_3_, 500 MHz)% *cis* ~ 80 with 60% **C**_**ss**_ incorporation. SEC (CHCl_3_) *M*_n_ = 29.0 kDa, *M*_w_ = 71.7 kDa, *Ð*_M_ = 2.5. DSC: *T*_g_ = −12.2 °C. TGA: *T*_d_ = 342 °C. Tensile tests: *E*_*0*_ = 12.0 ± 1.4 MPa, *ε*_break_ = 2077 ± 78%, UTS = 20.5 ± 0.6 MPa.

100% **C**_**ss**_: The polymer was prepared by the general procedure described above but with 1 eq of C_ss_. ^1^H NMR (CDCl_3_, 500 MHz)% *cis* ~ 80 with 100% **C**_**ss**_ incorporation. SEC (CHCl_3_) *M*_n_ = 62.0 kDa, *M*_w_ = 86.8 kDa, *Ð*_M_ = 1.4. DSC: *T*_g_ = −12.6 °C. TGA: *T*_d_ = 358 °C. Tensile tests: *E*_*0*_ = 4.6 ± 0.3 MPa, *ε*_break_ = 1886 ± 147%, UTS = 8.4 ± 1.9 MPa.

### Instrumental methods

#### Nuclear magnetic resonance spectroscopy

^1^H NMR spectra were acquired on a 500 MHz Bruker NMR spectrometer at 298 K in deuterated chloroform. 16 scans were acquired with a 3-second relaxation delay.

#### Size exclusion chromatography

Molecular weight was determined by SEC using an EcoSEC HLC-8320GPC (Tosoh Bioscience LLC, King of Prussia, PA) with a TSKgel GMH_HR_-M mixed bed column, refractive index (RI) detector and poly(styrene) standards. ~3 mg/mL samples were prepared using HPLC-grade chloroform (amylene-stabilized) for injection with a 0.5 mL/min flow rate at 40 °C.

#### Thermogravimetric analysis

Degradation temperatures for each of the copolymers were determined using TGA on a TA Instruments Discovery TGA550 (TA Instruments–Waters L.L.C, New Castle, DE). 5–10 mg samples were used with a ramp rate of 20 C/min from room temperature to 800  °C under N_2_ atmosphere. Degradation temperature was determined from the onset of the step transition (5% degradation).

#### Differential scanning calorimetry

Thermal transitions (*T*_g_, *T*_m_, and *T*_c_) were determined from the centers of the transitions acquired by DSC (first cooling and second heating cycle) on a TA Instruments Discovery DSC250 (TA Instruments–Waters L.L.C., New Castle, DE) using Trios Software Analysis. Unless otherwise noted, 5–10 mg samples were used with a ramp rate of 10  °C/min for both the heating and cooling cycles.

#### Variable cooling DSC

Variable cooling DSC experiments were performed to investigate the effect of cooling rate on crystallization on samples between 5 and 8 mg. The heating ramp rate was maintained at 10 °C/min across the same temperature distribution for each sample. Then various cooling rates were set to examine how *T*_c_ shifted as a result. Three control experiments were performed to establish the validity of these results. The first was a 4 cycle DSC experiment with 10 °C/min heating and cooling rate which confirmed there was no shift in T_c_ because of thermally cycling the material. The second control experiment was another DSC experiment to establish the reproducibility of the *T*_c_ shift. The polymer was cooled at some slow-cooling rate followed by a set heating cycle then cooled quickly. After the next heating cycle, it was cooled at the same slow-cooling rate to which showed no difference in *T*_c_ shift between the two slow-cooling cycles. Lastly, SEC was performed on the DSC-cycled samples with an uncycled control to confirm there was minimal thermal degradation. See Supplementary Fig. 24 for the validation experiments.

#### Compression molding

Melt press films were prepared on a bubble magic 5” × 5” pneumatic heat press (70 psi) between Teflon sheets, poly(acrylic acid) spin-coated Kapton sheets, or Mefine FEP film 10–20 °C above their melting temperature (as determined by DSC). Fast cooling was performed by removing pressed film and placing between two cold metal plates on the benchtop until cool. Slow-cooling was performed by setting the instrument to room temperature and maintaining pressure until temperature was below 40 °C. Preparation of barrier films was carried out as follows: the pristine polymer was pressed 5–10 minutes to flatten 20 °C above the T_m_ between thick Teflon sheets. This crude film was cooled quickly on the benchtop under heavy metal plates then used to fill a Kapton insert and pressed 10 minutes at the same temperature. After quick cooling, this film + insert was then pressed between mirrored metal plates and Mefine FEP film for 10 minutes and allowed to cool at the rate of the instrument to 40 °C or lower.

#### Contact angle

A ramé-hart instrument co. contact angle goniometer was used with DROPimage Pro software to calculate and image the contact angles of 5 μL of water on the polymer films. DROPimage Pro fits the shape of the droplet on the polymer film and measures the tangent angle between the solid-liquid interface.

#### Tensile testing

Dogbones were punched from 0.5-1 mm thick films using a Pioneer Die-tecs die following ASTM D-638-V. Tensile Instron universal testing machine at room temperature with a 1 kN load cell at 10 mm/min displacement rate. Young’s Modulus was determined by dividing the stress by the strain% at 5% strain for each replicate. Ultimate tensile strength was determined from the maximum stress observed, and elongation at break was determined from the strain% observed at break.

#### X-ray diffraction (XRD)

XRD was performed using a Panalytical X-Pert PRO MRD HR XRD system in a two-theta analysis configuration with a Cu K-alpha source, ½° divergence slit, and Xe proportion detector. The diffraction pattern was collected from 10–80° with a 0.05 step size at 1 s/step.

### Method for calculating lamellar thicknesses

Lamellar thicknesses were determined from XRD of 15–25% C_ss_ by the Debye-Scherrer equation:

$$l=\frac{0.9\lambda }{{FWHM}\cos \frac{\theta }{2}}$$

#### SAXS and WAXS

SAXS and WAXS measurements were performed on a SAXSLab Ganesha system with a Cu 50 kV Xenocs Genix ULD SL X-ray point source and a 170 μm pixel-spaced, single-photon counting Dectris Pilatus 300 k 20 Hz detector. The measurements were performed using a 2 slit configuration where the distance between the sample and the detector was approximately 101, for WAXS and 1041 mm for SAXS. One dimensional (1D) averaging of SAXS data was carried out by averaging across all azimuths with linear *x* axis binning. Spectra-by-spectra reduction and corrections for sample transmission, sample thickness and absolute intensity factor were all included as part of 1D averaging using saxsgui software v2.23.33. To accurately determine the momentum transfer corresponding to the peak intensities in SAXS/WAXS data, the patterns were modeled using Fityk software. A spline was used to flatten the baseline and gaussian distributions (7 for WAXS, 2 for SAXS) were used to model the pattern by minimizing the residual. The degree of crystallinity was determined from these fitting parameters by taking dividing the gaussian areas corresponding to the crystalline regimes by the crystalline and amorphous gaussian areas.

#### Atomic force microscopy (AFM)

AFM was performed in tapping mode with Budget Sensors Tap300GD-G tips (resonant frequency = 300 kHz, force constant = 40 N/m) on an Asylum Cypher ES Environmental atomic force microscope.

#### Dynamic vapor sorption

DVS was performed on a TA Q5000 SA sorption analyzer with 5–20 mg of polymer film at 25 °C and 0% relative humidity (RH) for 10 minutes. RH was then ramped at 25 °C 0.5% min^−1^ from 0% RH to 97.5% RH. 97.5% RH was maintained for 120 minutes after this point. PET control was provided by TA instruments with DHR RH accessory.

### Molecular dynamics simulations methods

1. All simulations were performed as follows: Randomly paced *N*_chain_ copolymer chains inside a cubic simulation box with size *L* = 200*σ.*
2. Changed all non-bonded interactions between coarse-grained beads into WCA potential (*ε*_LJ_ = 1.0 *k*_B_*T* and *r*_cut_ = 2^1/6^ *σ*), followed by the system relaxation under NVT ensemble conditions (a1) for duration 10000 τ_LJ_.
3. Gradually decreased the simulation box to achieve the density 0.1 *σ*^−3^, and relaxed the system for another 1000 τ_LJ_.
4. Changed non-bonded interaction between coarse-grained beads to the parameters shown in Table 1, and increased the system temperature to 2.0 in energy units followed by the system relaxation lasting 1000 τ_LJ_.
5. Switched to NPT ensemble (a2) with the temperature *T* = 2.0 and relaxed the system for additional 5000 τ_LJ_.
6. After the system was fully relaxed at *T* = 2.0 under NPT ensemble conditions, we performed series of annealing cycles to reach an equilibrium. In each annealing cycle, the simulation temperature T was decreased from *T*_max_ = 2.0 to *T*_min_ = 1.0 during simulation run lasting 10000 τ_LJ_. After the first annealing cycle was completed, the second cycle began with *T*_max_ = 1.9 and then repeat the cycle until *T*_min_ = 1.0.
7. After completing the annealing cycles, a simulation run at *T* = 1.0 was performed for data collection.

### Histological methods

Films were prepared for implantation by blade-coating 20%wt/wt polymer solutions in chloroform on a PTFE substrate using an EZ Coater EC-200 (ChemInstruments). Uniform films 100 µm and 200 µm thick were air dried for 24 hours at ambient temperature before vacuum drying for 48 h at ambient temperature. 8 mm diameter biopsy punches were used to obtain sample discs from the prepared films then sterilized with EtO (Ethylene Oxide) before implantation.

### Implantation and tissue processing

The investigational Protocol #A168-20-8 for this study was reviewed and approved by the Duke Institutional Animal Care and Use Committee (IACUC). Sprague-Dawley rats (*n* = 8) of equal sexes (*n* = 4 each sex) were used in each experimental group. Prior to the surgical procedure, the rats received an anesthetic drug cocktail (ketamine, xylazine, acepromazine, 29.6:5.95:0.53 mg/kg respectively). Isoflurane (2.0%) was administered to each rat through a nose-cone during the surgical procedure to maintain an anesthetized state. Four dorsal incisions (1 cm in length) equidistance apart from the spine were created using scalpel and hemostats were used to create a subcutaneous pocket followed by implantation of the polymer discs. The incisions were then closed with Michel clips. Cage side clinical observations were performed regularly with particular attention paid to the incision site.

At preset timepoints, animals were euthanized by intraperitoneal injection (1 mL) of pentobarbital after sedation with isoflurane (2%) in oxygen. Copolymer disc samples and surrounding tissue were collected postmortem, fixed in neutral buffered formalin (NBF) (10%), and embedded in paraffin for processing. Embedded samples were sectioned (5 μm thick) and mounted on microscope slides. Substrates were stained with hematoxylin and eosin (H&E) and then fixed in a DPX histology mount. Substrates were quantified on a modified scoring system outlined by the International Organization for Standardization (ISO 10993-6 Annex E) by a board-certified veterinary pathologist. The numbers of inflammatory cells were estimated by light microscopy (×400) and a score was assigned for each inflammatory cell type. Scoring was performed with, 0 = absent, 1 = minimal, 2 = mild, 3 = moderate, 4 = severe, as the scale (*n* = 4).

### Reporting summary

Further information on research design is available in the Nature Portfolio Reporting Summary linked to this article.

### Supplementary information

Supplementary Information

Peer Review File

Reporting Summary

## Data Availability

Data will be made available upon request to the authors. Data for Fig. 8 was manually compiled from homogenous, nonproprietary polymer (not reinforced or filled) entries from Matweb.com^38^.
