# Supplementary material for: Resorbable barrier polymers for flexible bioelectronics
Source: Nat Commun. 2023 Nov 11;14:7299. doi: 10.1038/s41467-023-42775-5 (PMC10638316; doi:10.1038/s41467-023-42775-5)
Supplement: Supplementary file 1 — Supplementary Information [file 41467_2023_42775_MOESM1_ESM.pdf]

# Supplementary Information

# Resorbable Barrier Polymers for Flexible Bioelectronics

Samantha M. McDonald[1], Quansan Yang[2,3], Yen-Hao Hsu[1], Shantanu P. Nikam[1], Ziying Hu[3], Zilu Wang[4], Darya Asheghali[1], Tiffany Yen[1], Andrey V. Dobrynin[4], John A. Rogers[2,3,5,6*] and Matthew L. Becker[1,7,8,9*]

[1] Department of Chemistry, Duke University, Durham, NC 27708, United States

[2] Department of Mechanical Engineering, Northwestern University, Evanston, IL, 60208 USA

[3] Querrey Simpson Institute for Bioelectronics, Northwestern University, Evanston, IL, 60208 USA

[4] Department of Chemistry, University of North Carolina-Chapel Hill, Chapel Hill, NC 27599, USA

[5] Department of Biomedical Engineering and Neurological Surgery, Northwestern University, Evanston, IL, 60208 USA

[6] Department of Materials Science and Engineering, Northwestern University, Evanston, IL, 60208 USA

[7] Thomas Lord Department of Mechanical Engineering and Materials Science, Duke University, Durham, NC 27708, USA

[8]Department of Biomedical Engineering, Duke University, Durham, NC 27708, USA

[9]Department of Orthopedic Surgery, Duke University, Durham, NC 27708, USA

**Corresponding Authors**

**Matthew L. Becker, PhD**
Hugo L. Blomquist Distinguished Professor
Duke University
Department of Chemistry, Thomas Lord Department of Mechanical Engineering & Material Science,
Department of Biomedical Engineering and Department of Orthopaedic Surgery
308 Research Drive
Durham, NC 27708
(919) 681-3812
matthew.l.becker@duke.edu

**John A. Rogers, PhD**
Louis Simpson and Kimberly Querrey Professor
Northwestern University
Querrey Simpson Institute for Bioelectronics
Departments of Mechanical Engineering, Material Science and Engineering, Biomedical Engineering
Evanston, IL, 60208, USA
jrogers@northwestern.edu

## CONTENTS

**Supplementary Figure 1.** The $^{1}$H NMR spectrum of 0% **$C_{ss}$**……………………………………………………4
**Supplementary Figure 2.** SEC chromatogram of 0% **$C_{ss}$** ……………………………………………………5
**Supplementary Figure 3.** The $^{1}$H NMR spectrum of 10% **$C_{ss}$** ……………………………………………6
**Supplementary Figure 4.** SEC chromatogram of 10% **$C_{ss}$** ……………………………………………………7
**Supplementary Figure 5.** The $^{1}$H NMR spectrum of 15% **$C_{ss}$** ……………………………………………8
**Supplementary Figure 6.** SEC chromatogram of 15% **$C_{ss}$** ……………………………………………………9
**Supplementary Figure 7.** The $^{1}$H NMR spectrum of 20% **$C_{ss}$** ……………………………………………10
**Supplementary Figure 8.** SEC chromatogram of 20% **$C_{ss}$** ……………………………………………………11
**Supplementary Figure 9.** The $^{1}$H NMR spectrum of 25% **$C_{ss}$** ……………………………………………12
**Supplementary Figure 10.** SEC chromatogram of 25% **$C_{ss}$** …………………………………………………13
**Supplementary Figure 11.** The $^{1}$H NMR spectrum of 30% **$C_{ss}$** …………………………………………14
**Supplementary Figure 12.** SEC chromatogram of 30% **$C_{ss}$** …………………………………………………15
**Supplementary Figure 13.** The $^{1}$H NMR spectrum of 40% **$C_{ss}$** …………………………………………16
**Supplementary Figure 14.** SEC chromatogram of 40% **$C_{ss}$** …………………………………………………17
**Supplementary Figure 15.** The $^{1}$H NMR spectrum of 50% **$C_{ss}$** …………………………………………18
**Supplementary Figure 16.** SEC chromatogram of 50% **$C_{ss}$** …………………………………………………19
**Supplementary Figure 17.** The $^{1}$H NMR spectrum of 60% **$C_{ss}$** …………………………………………20
**Supplementary Figure 18.** SEC chromatogram of 60% **$C_{ss}$** …………………………………………………21
**Supplementary Figure 19.** The $^{1}$H NMR spectrum of 100% **$C_{ss}$** ………………………………………22
**Supplementary Figure 20.** SEC chromatogram of 100% **$C_{ss}$** ………………………………………………23
**Supplementary Figure 21.** Overlapped TGA data of all synthesized polymers………………………………24
**Supplementary Figure 22.** Overlapped DSC thermograms of all synthesized polymers ……………………24
**Supplementary Figure 23.** Stress vs. strain curves for all synthesized polymers …………………………25
**Supplementary Figure 24.** Control experiments for Variable Cooling Rate DSC Experiments……………26
**Supplementary Figure 25.** *In vitro* degradation studies……………………………………………………27
**Supplementary Figure 26.** Variable cooling rate DSC of 60% $C_{ss}$ $C_{ss}$ ……………………………………27
**Supplementary Figure 27.** VT-WAXS experiments of 60% $C_{ss}$………………………………………………28
**Supplementary Figure 28.** XRD of 10-25% $C_{ss}$……………………………………………………………28
**Supplementary Figure 29.** Representative Hoffman-Weeks extrapolation…………………………………29
**Supplementary Figure 30.**2D Scattering Patterns for 20% Css…………………………………………………29
**Supplementary Figure 31.** Partial Charge Distributions for Monomer Sequences……………………………30
**Supplementary Figure 32.** Additional Scattering Functions for Other Copolymer Compositions ……………31
**Supplementary Figure 33.** Variable Temperature WAXS for 20% $C_{ss}$ Copolymers ……………………………32

**Supplementary Figure 34.** DSC Thermograms of Melt-Pressed 20% $C_{ss}$ ……………………………..…..…..33
**Supplementary Figure 35.** Molecular Weight Study of 20% Css Copolymers ………………………..…..…..33
**Supplementary Figure 36.** Stoichiometry and alkene conformation over time in 20% Css Polymerization…..…..34
**Supplementary Figure 37.** Proposed Mechanism for Thiol-yne Copolymerization ……………………….….34
**Supplementary Figure 38.** Histopathology of 100 μm thick vs. 200 μm thick films ……………………...…….35
**Supplementary Table 1.** Histology scoring of $C_{ss}$ *in vivo* compatibility study………… …..……..…………..35
**Supplementary Table 2.** Simulated Equilibrium Phase Structure for $C_{ss}$ Coolymer Melts ……………………….36
**Supplementary Methods.** …………………...………….…… …………...…………………….…………….36
**References**……….……………………………………………………………………...………...…......…..36

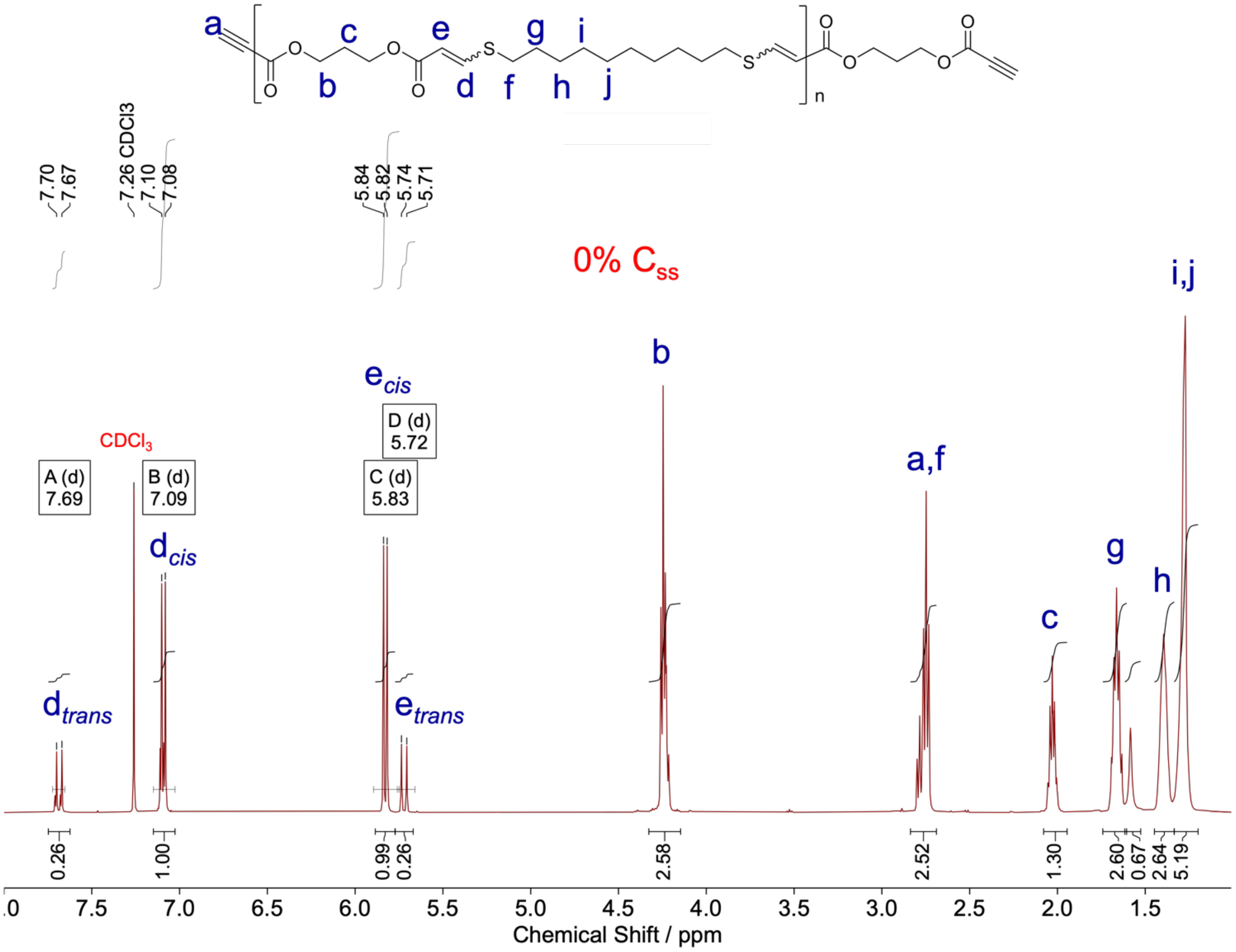


**Supplementary Figure 1.** The $^{1}$H NMR spectra of 0% $\mathbf{C_{SS}}$ shows the 81% *cis* content for the thiol-yne step-growth polymer. *Cis*- content can be calculated by the *J* coupling constants of the respective resonances. Resonance *f, g, h, i,* and *j* are from the dithiol monomer ($\mathbf{C_{10S}}$) and resonance *a, b, and c* are from the 1,3-propane dipropiolate monomer ($\mathbf{C_{3A}}$). The polymer reveals *cis* %/ *trans* % = 81 %: 19 % in $CHCl_3$ with 1 mol% DBU ($CDCl_3$, 500 MHz).

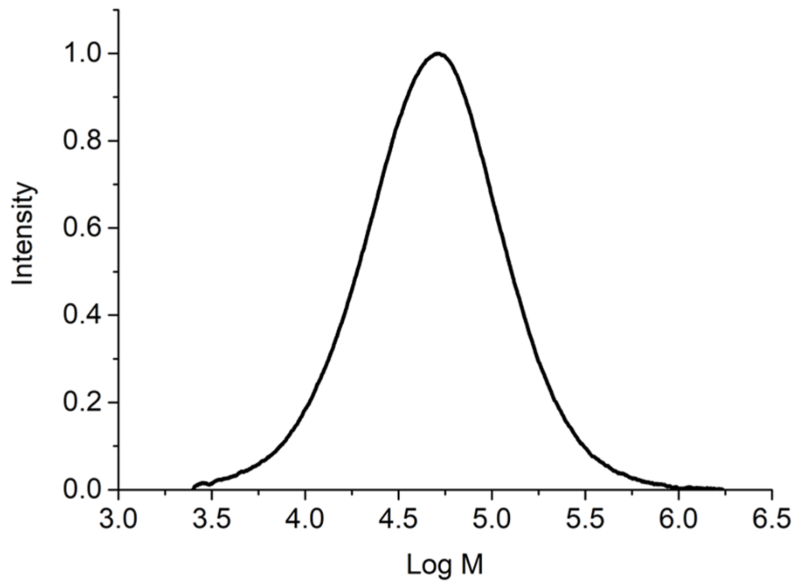


**Supplementary Figure 2.** SEC chromatogram of 0% $\mathbf{C}_{\mathrm{SS}}$ of thiol-yne step-growth polymer; $M_n$ = 32.5 kDa, $M_w$ = 72.8 kDa, $Đ_M$ = 2.24 (SEC $CHCl_3$, based on PS standards).

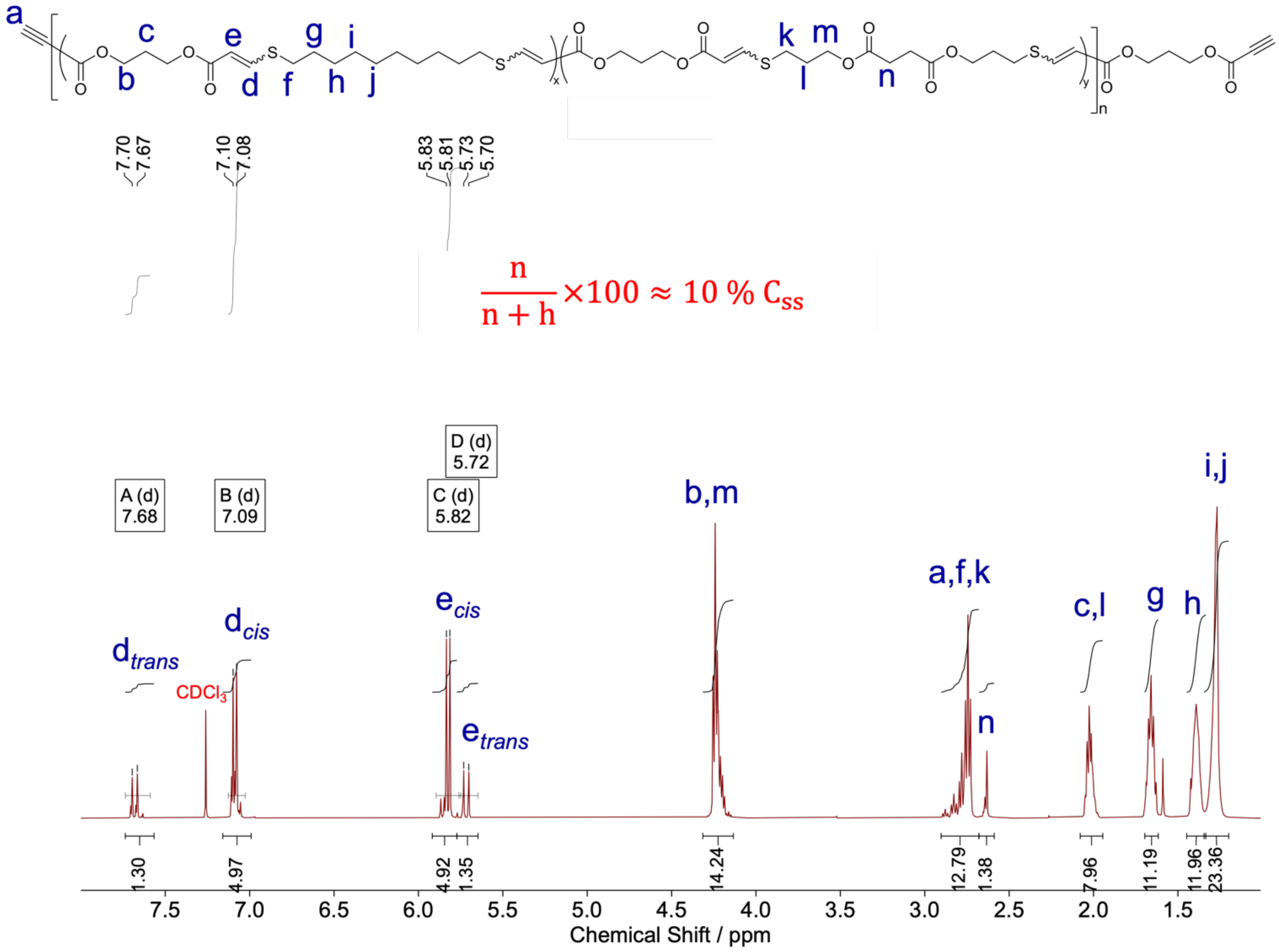


**Supplementary Figure 3.** The $^{1}$H NMR spectra of 10% $\mathbf{C_{SS}}$ shows the 80% *cis* content for the thiol-yne step-growth copolymer with 10% incorporation of $\mathbf{C_{SS}}$ in $CHCl_3$ with 1 mol% DBU. The ratio of resonance *n* (from $\mathbf{C_{SS}}$) to resonance *h* (from $\mathbf{C_{10S}}$) displays 10%: 90% that affords a polymer 10% incorporation of $\mathbf{C_{SS}}$ ($CDCl_3$, 500 MHz).

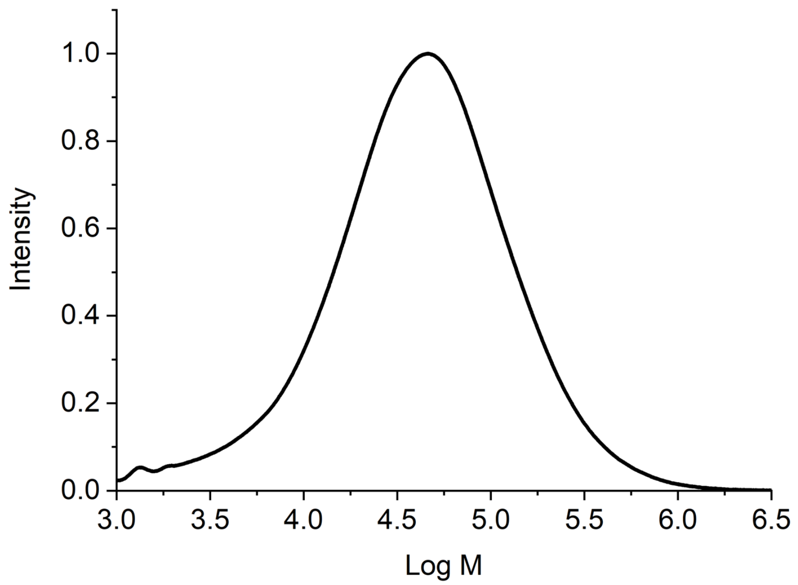


**Supplementary Figure 4.** SEC chromatogram of 10% $\mathbf{C}_{SS}$ of thiol-yne step-growth polymer; $M_n$ = 32.1 kDa, $M_w$ = 81.3 kDa, $Đ_M$ = 2.51. (SEC $CHCl_3$, based on PS standards).

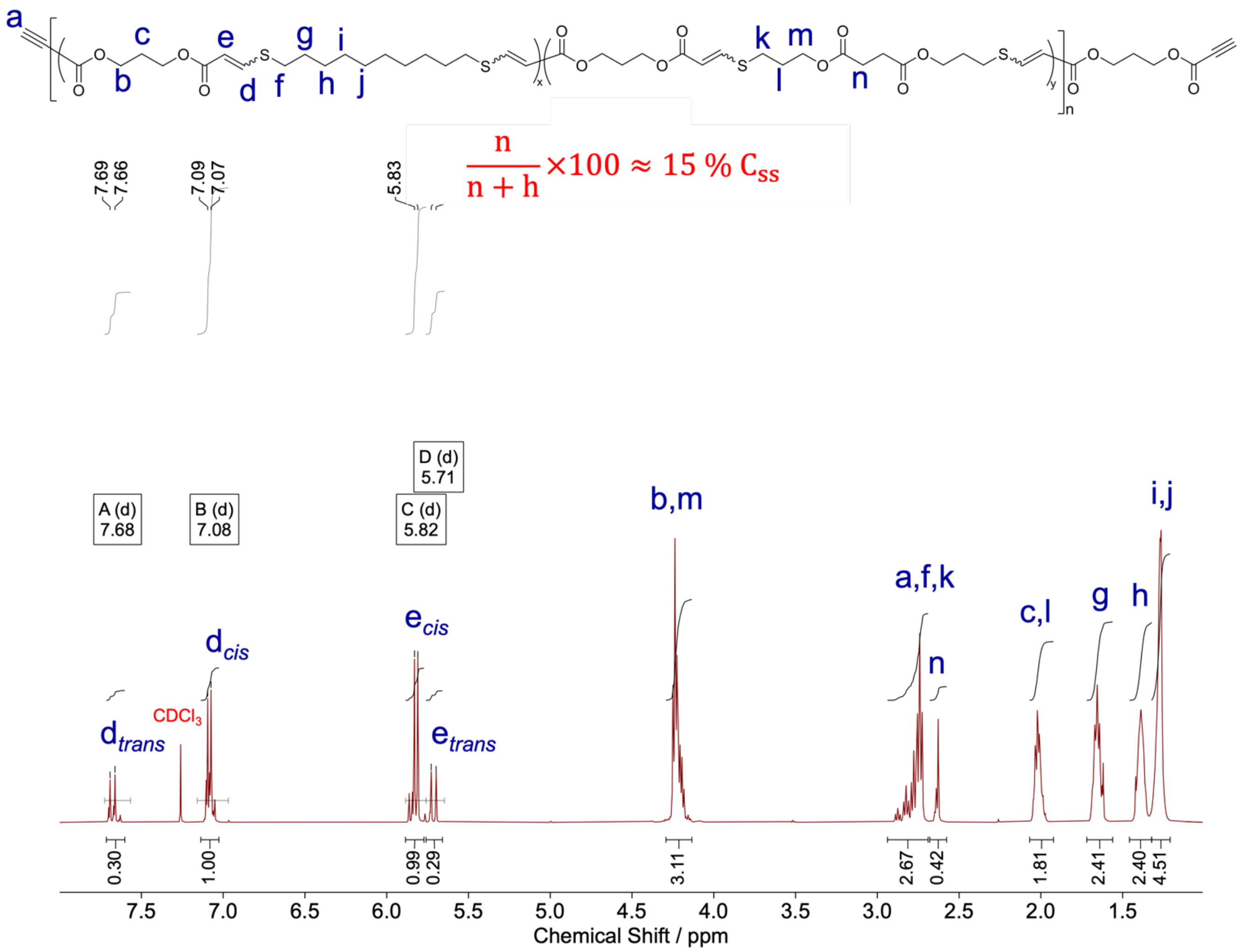


**Supplementary Figure 5.** The $^{1}$H NMR spectra of 15% **C**$_{SS}$ shows the 80% *cis* content for the thiol-yne step-growth copolymer with 15% incorporation of **C**$_{SS}$ in $CHCl_3$ with 1 mol% DBU. The ratio of methylene resonance *n* (from **C**$_{SS}$) to methylene resonance *h* (from **C**$_{10S}$) displays 15%: 85% that affords polymer 15% incorporation of **C**$_{SS}$ ($CDCl_3$, 500 MHz).

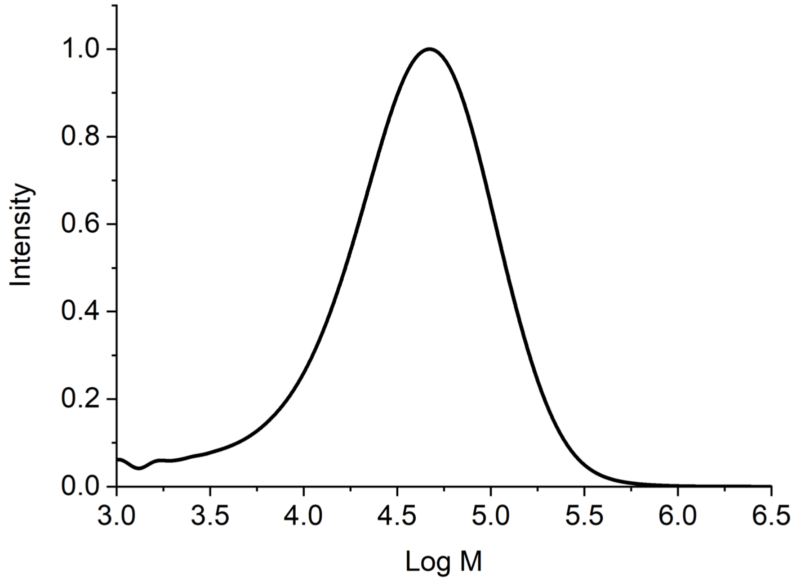


**Supplementary Figure 6.** SEC chromatogram of 15% **C**$_{SS}$ of thiol-yne step-growth polymer; $M_n$ = 23.6 kDa, $M_w$ = 54.5 kDa, $Đ_M$ = 2.30. (SEC $CHCl_3$, based on PS standards).

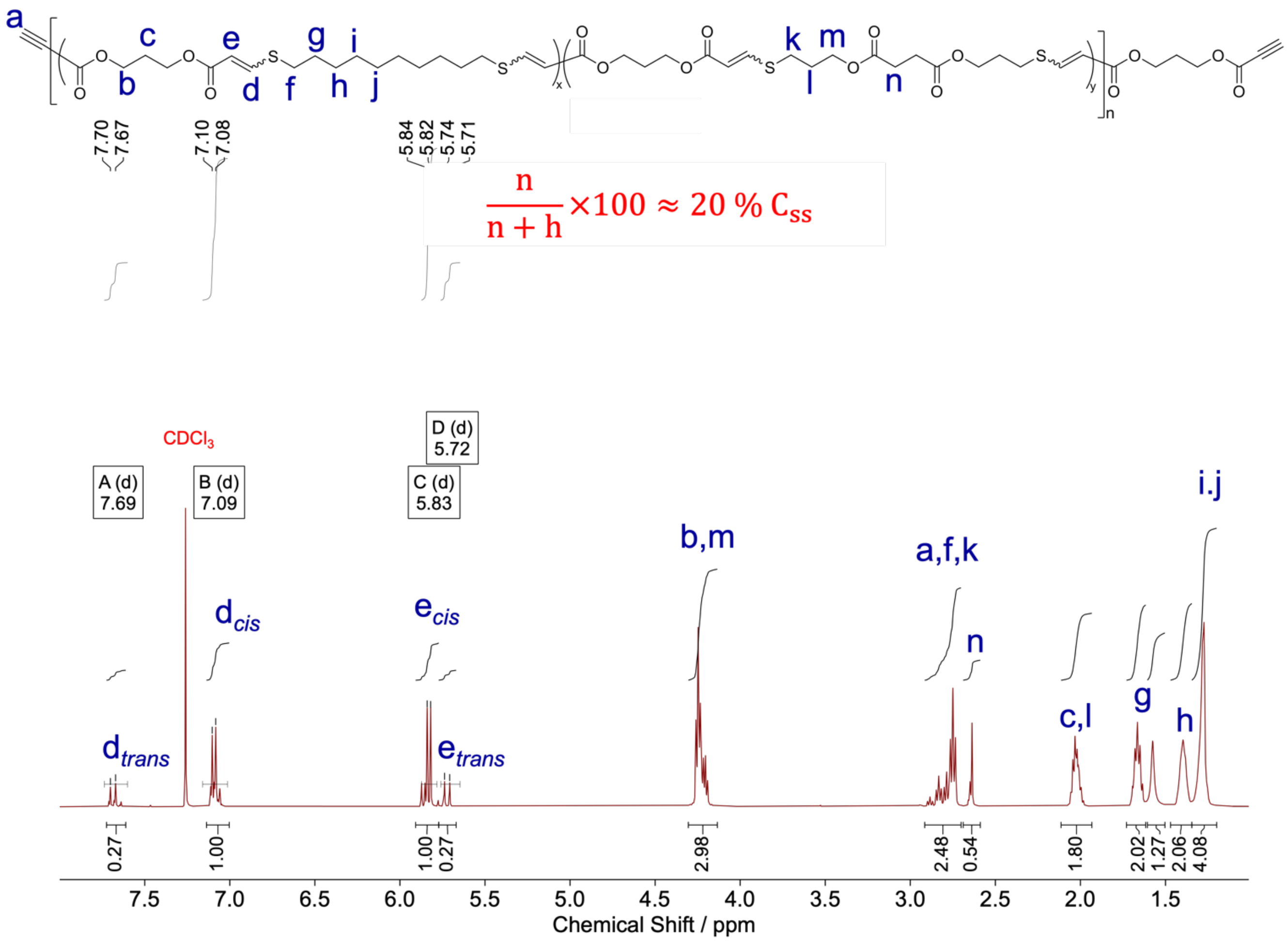


**Supplementary Figure 7.** The $^{1}$H NMR spectra of 20% $\mathbf{C_{SS}}$ shows the 80% *cis* content for the thiol-yne step-growth copolymer with 20% incorporation of $\mathbf{C_{SS}}$ in $CHCl_3$ with 1 mol% DBU. The ratio of methylene resonance *n* (from $\mathbf{C_{SS}}$) to methylene resonance *h* (from $\mathbf{C_{10S}}$) displays 20%: 80% that affords polymer with 20% incorporation of $\mathbf{C_{SS}}$ ($CDCl_3$, 500 MHz).

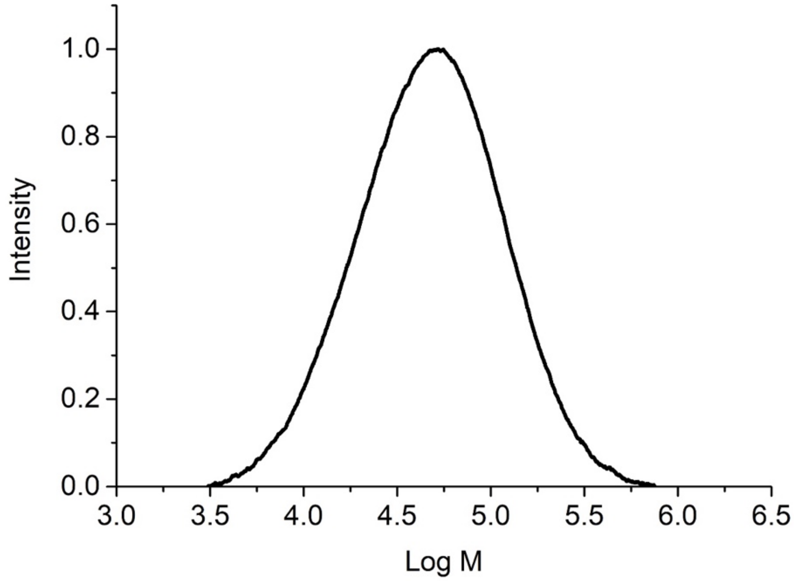


**Supplementary Figure 8.** SEC chromatogram of 20% $\mathbf{C}_{SS}$ of thiol-yne step-growth polymer with 20% incorporation of $\mathbf{C}_{SS}$; $M_n$ = 32.6 kDa, $M_w$ = 68.6 kDa, $Đ_M$ = 2.10 (SEC $CHCl_3$, based on PS standards).

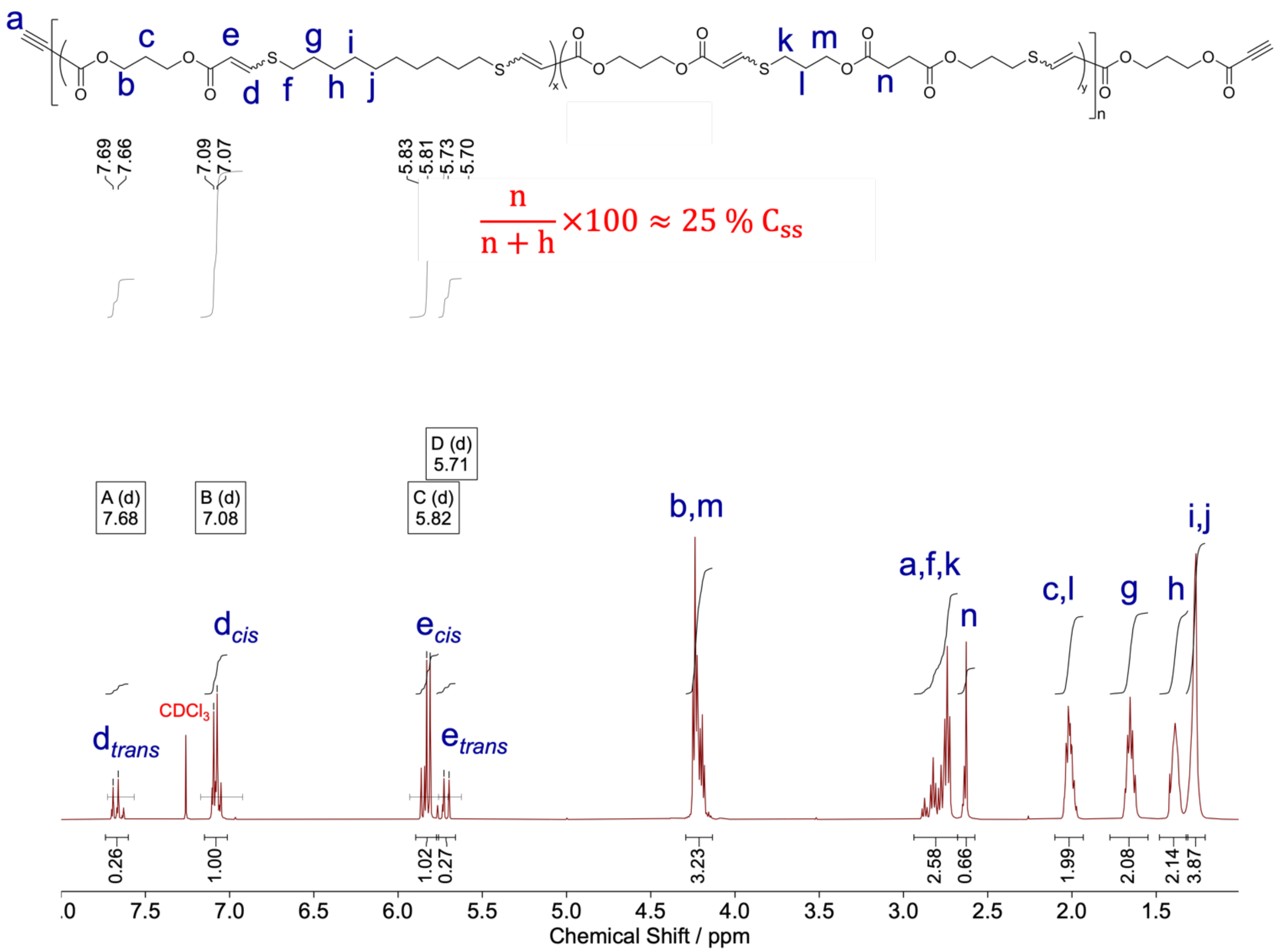


**Supplementary Figure 9.** The $^{1}$H NMR spectra of 25% $\mathbf{C_{SS}}$ shows the 80% *cis* content for the thiol-yne step-growth copolymer with 25% incorporation of $\mathbf{C_{SS}}$ in $CHCl_3$ with 1 mol% DBU. The ratio of methylene resonance *n* (from $\mathbf{C_{SS}}$) to methylene resonance *h* (from $\mathbf{C_{10S}}$) displays 25%: 75% that affords polymer with 25% incorporation of $\mathbf{C_{SS}}$ ($CDCl_3$, 500 MHz).

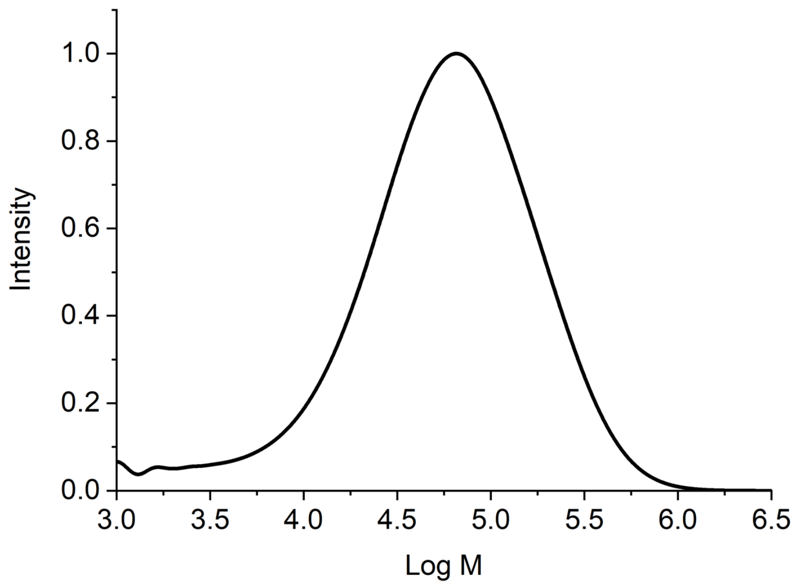


**Supplementary Figure 10.** SEC chromatogram of 25% **C$_{SS}$** of thiol-yne step-growth polymer; $M_n$ = 34.0 kDa, $M_w$ = 89.0 kDa, $Đ_M$ = 2.62. (SEC $CHCl_3$, based on PS standards).

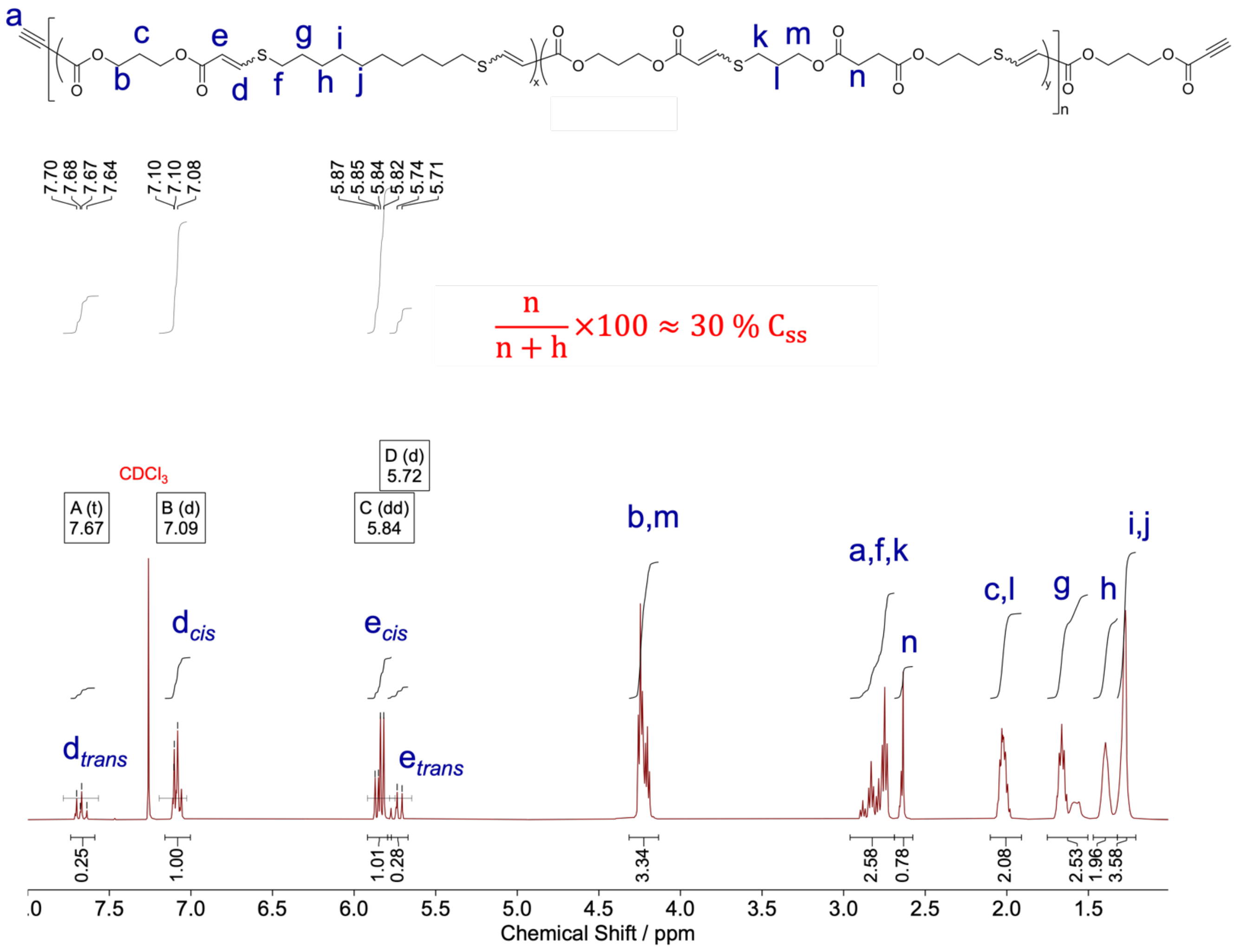


**Supplementary Figure 11.** The $^{1}H$ NMR spectra of 30% $\mathbf{C_{SS}}$ shows the 80% *cis* content for the thiol-yne step-growth copolymer with 30% incorporation of $\mathbf{C_{SS}}$ in $CHCl_3$ with 1 mol% DBU. The ratio of methylene resonance *n* (from $\mathbf{C_{SS}}$) to methylene resonance *h* (from $\mathbf{C_{10S}}$) displays 30%: 70% that affords polymer with 30% incorporation of $\mathbf{C_{SS}}$ ($CDCl_3$, 500 MHz).

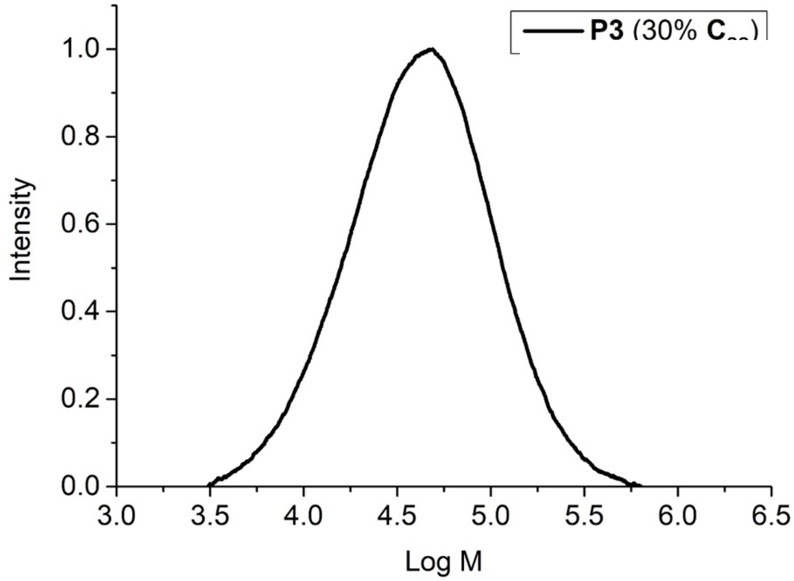


**Supplementary Figure 12.** SEC chromatogram of 30% $\mathbf{C}_{SS}$ of thiol-yne step-growth polymer with 30% incorporation of $\mathbf{C}_{SS}$; $M_n$ = 29.1 kDa, $M_w$ = 60.4 kDa, $Đ_M$ = 2.08 (SEC $CHCl_3$, based on PS standards).

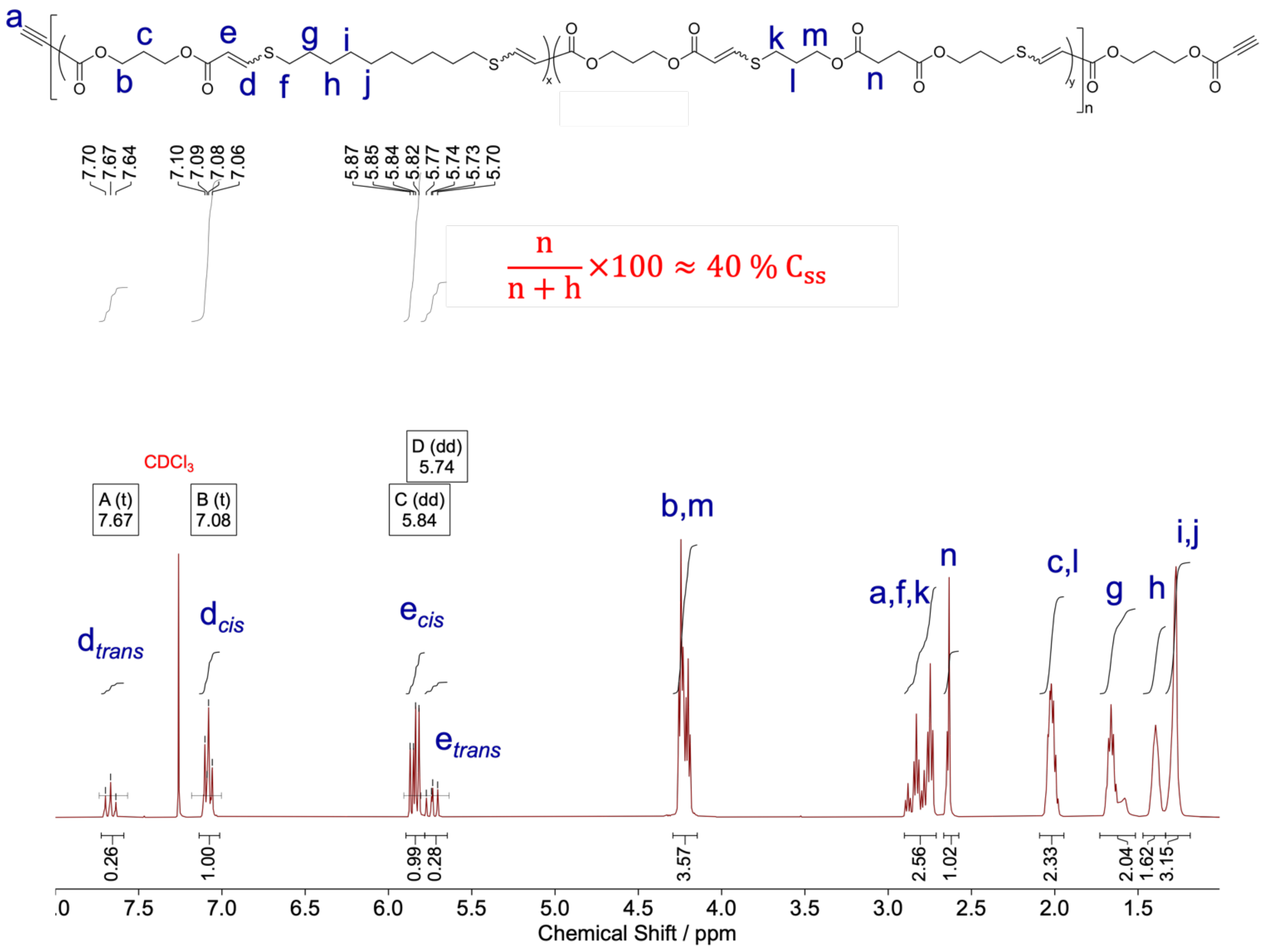


**Supplementary Figure 13.** The $^{1}$H NMR spectra of 40% $\mathbf{C_{SS}}$ shows the 80% *cis* content for the thiol-yne step-growth copolymer with 40% incorporation of $\mathbf{C_{SS}}$ in $CHCl_3$ with 1 mol% DBU. The ratio of methylene resonance *n* (from $\mathbf{C_{SS}}$) to methylene resonance *h* (from $\mathbf{C_{10S}}$) displays 40%: 60% that affords polymer with 40% incorporation of $\mathbf{C_{SS}}$ ($CDCl_3$, 500 MHz).

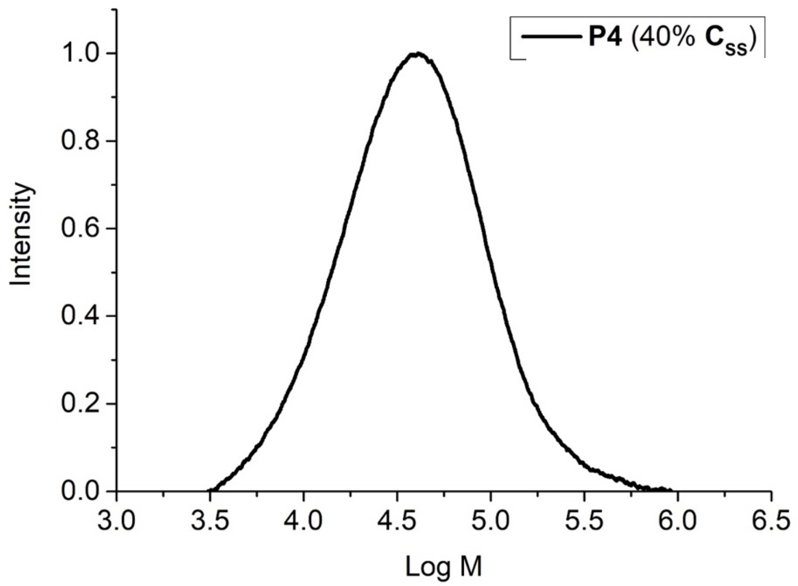


**Supplementary Figure 14.** SEC chromatogram of 40% $\mathbf{C}_{SS}$ of thiol-yne step-growth polymer with 40% incorporation of $\mathbf{C}_{SS}$; $M_n$ = 26.7 kDa, $M_w$ = 57.0 kDa, $Đ_M$ = 2.13 (SEC $CHCl_3$, based on PS standards).

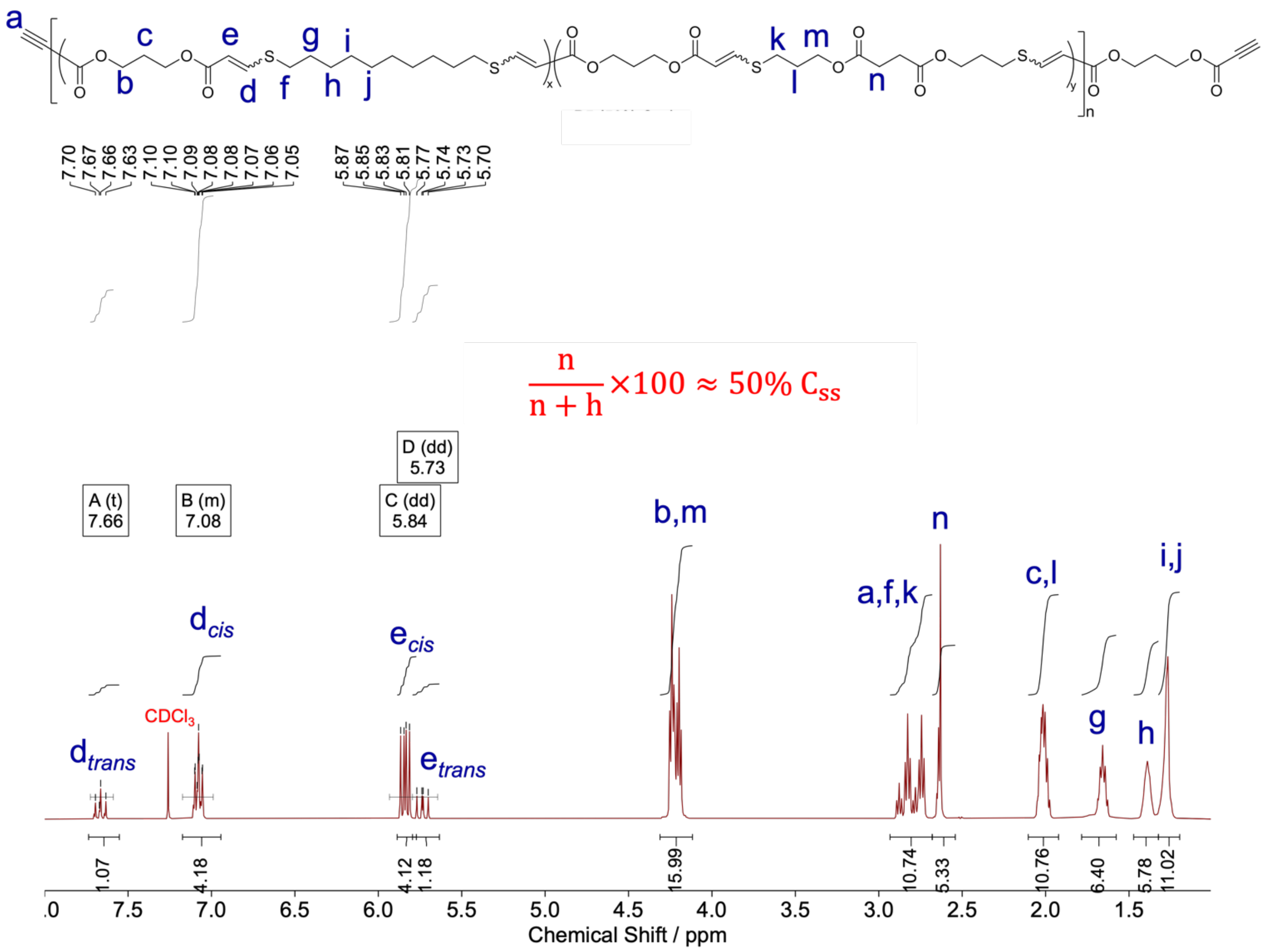


**Supplementary Figure 15.** The $^{1}$H NMR spectra of 50% $\mathbf{C_{SS}}$ shows the 80% *cis* content for the thiol-yne step-growth copolymer with 50% incorporation of $\mathbf{C_{SS}}$ in $CHCl_3$ with 1 mol% DBU. The ratio of methylene resonance *n* (from $\mathbf{C_{SS}}$) to methylene resonance *h* (from $\mathbf{C_{10S}}$) displays 49%: 51% that affords polymer with 49% incorporation of $\mathbf{C_{SS}}$ ($CDCl_3$, 500 MHz).

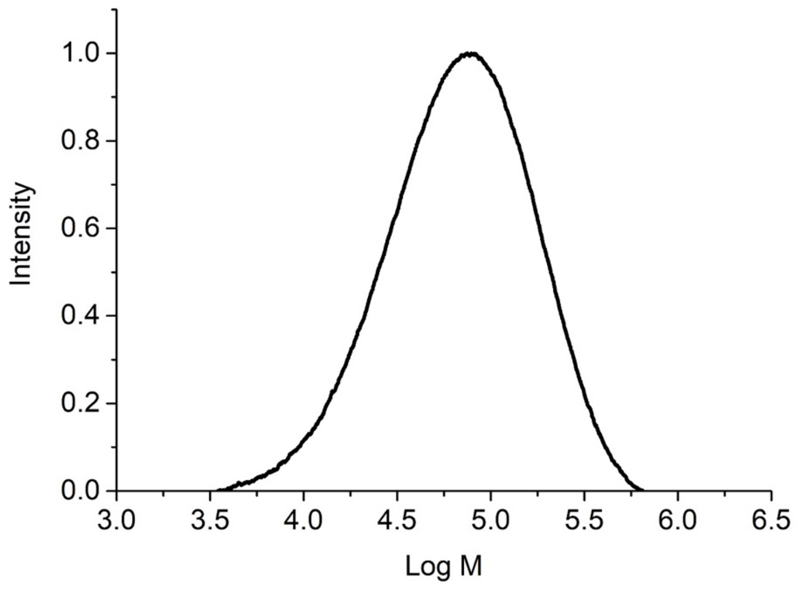


**Supplementary Figure 16.** SEC chromatogram of 50% $\mathbf{C}_{SS}$ of thiol-yne step-growth polymer with 50% incorporation of $\mathbf{C}_{SS}$; $M_n$ = 45.4 kDa, $M_w$ = 94.9 kDa, $Đ_M$ = 2.09 (SEC $CHCl_3$, based on PS standards).

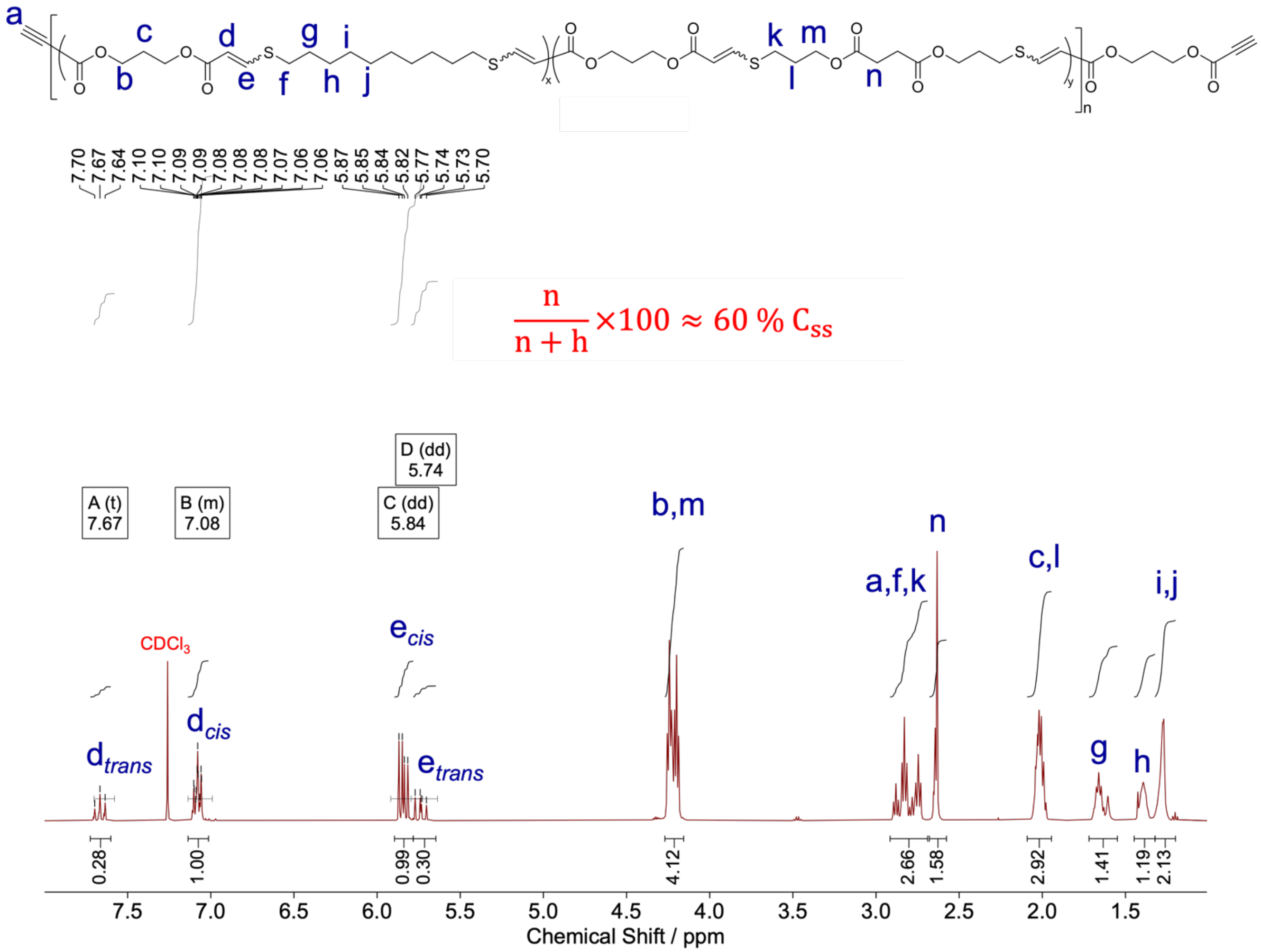


**Supplementary Figure 17.** The $^{1}$H NMR spectra of 60% $\mathbf{C_{SS}}$ shows the 80% *cis* content for the thiol-yne step-growth copolymer with 60% incorporation of $\mathbf{C_{SS}}$ in $CHCl_3$ with 1 mol% DBU. The ratio of methylene resonance *n* (from $\mathbf{C_{SS}}$) to methylene resonance *h* (from $\mathbf{C_{10S}}$) displays 60%: 40% that affords polymer with 60% incorporation of $\mathbf{C_{SS}}$ ($CDCl_3$, 500 MHz).

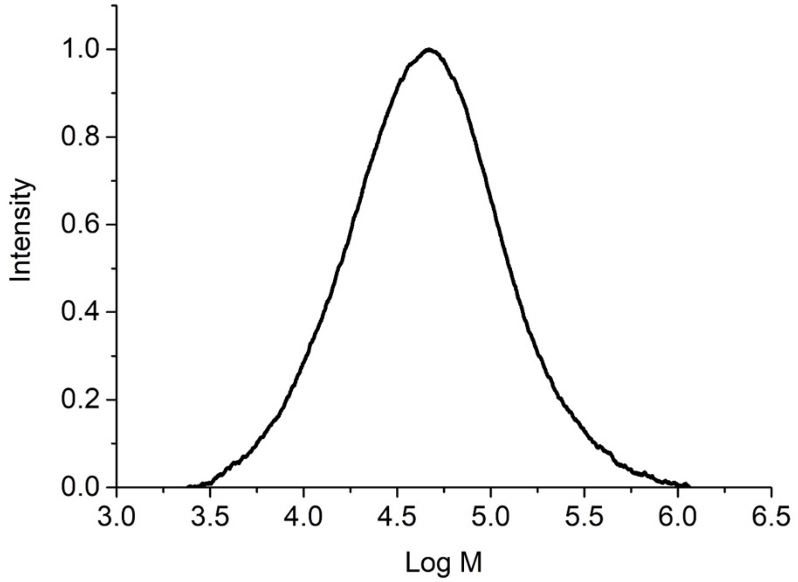


**Supplementary Figure 18.** SEC chromatogram of 60% $\mathbf{C}_{SS}$ of thiol-yne step-growth polymer with 60% incorporation of $\mathbf{C}_{SS}$; $M_n$ = 29.0 kDa, $M_w$ = 71.7 kDa, $Đ_M$ = 2.47 (SEC $CHCl_3$, based on PS standards).

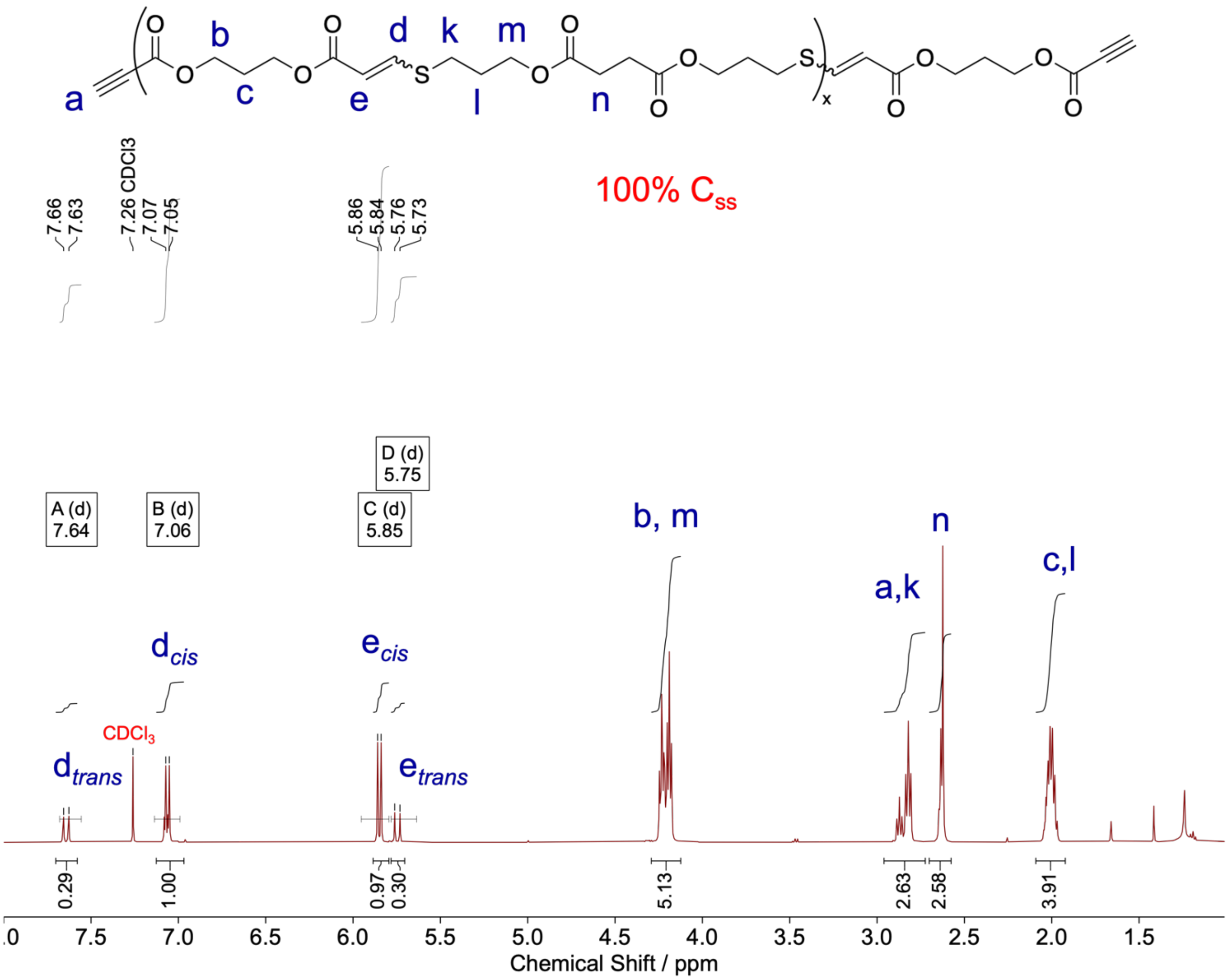


**Supplementary Figure 19.** The $^{1}$H NMR spectra of 100% **C$_{SS}$** shows the 80% *cis* content for the thiol-yne step-growth copolymer with 100% incorporation of **C$_{SS}$** in $CHCl_3$ with 1 mol% DBU.

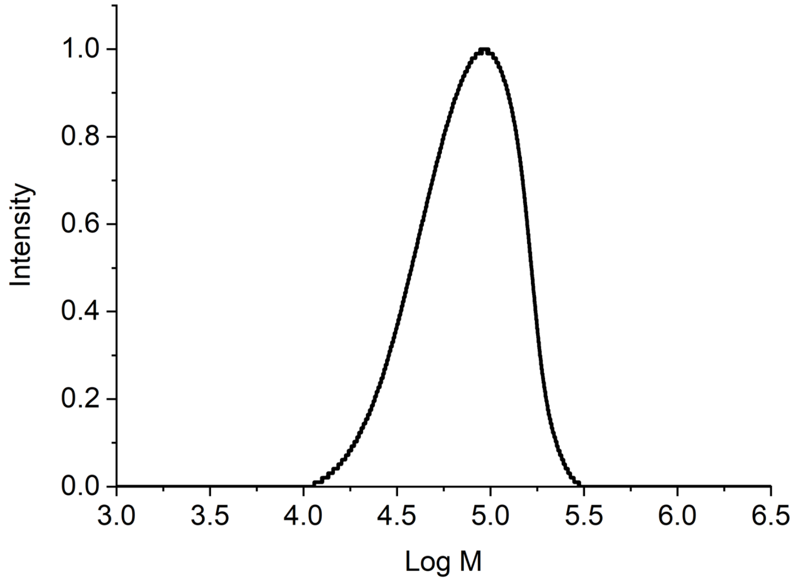


**Supplementary Figure 20.** SEC chromatogram of 100% $\mathbf{C}_{SS}$ of thiol-yne step-growth polymer with 100% incorporation of $\mathbf{C}_{SS}$; $M_n$ = 62.0 kDa, $M_w$ = 86.8 kDa, $Đ_M$ = 1.4 (SEC $CHCl_3$, based on PS standards).

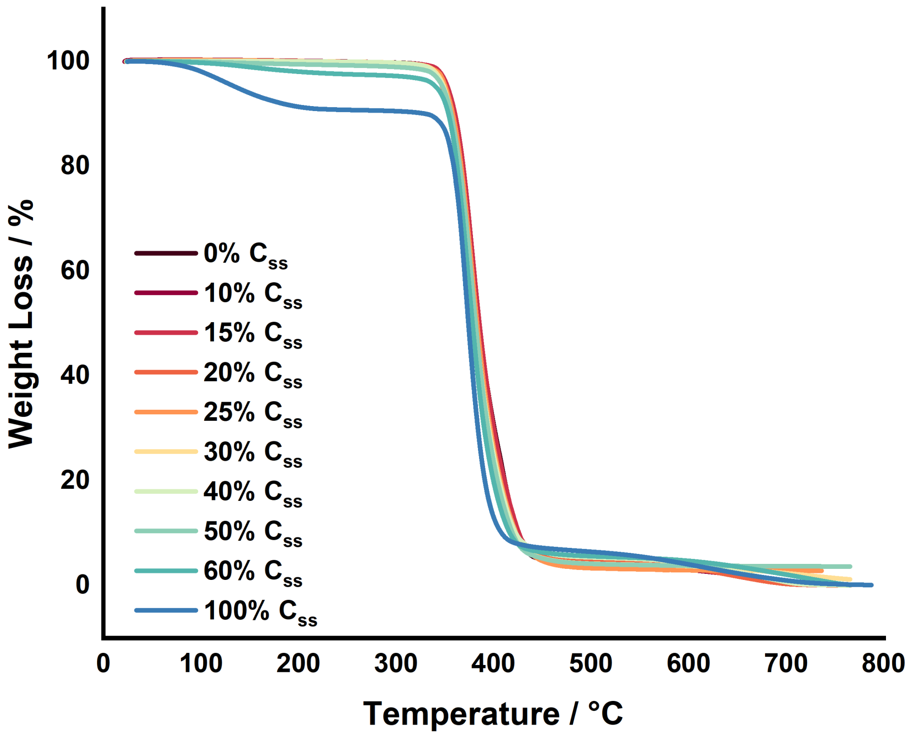


**Supplementary Figure 21.** Overlapped data of thermogravimetric analysis (TGA) of all synthesized polymers were performed to determine the degradation temperature and profile for each species.

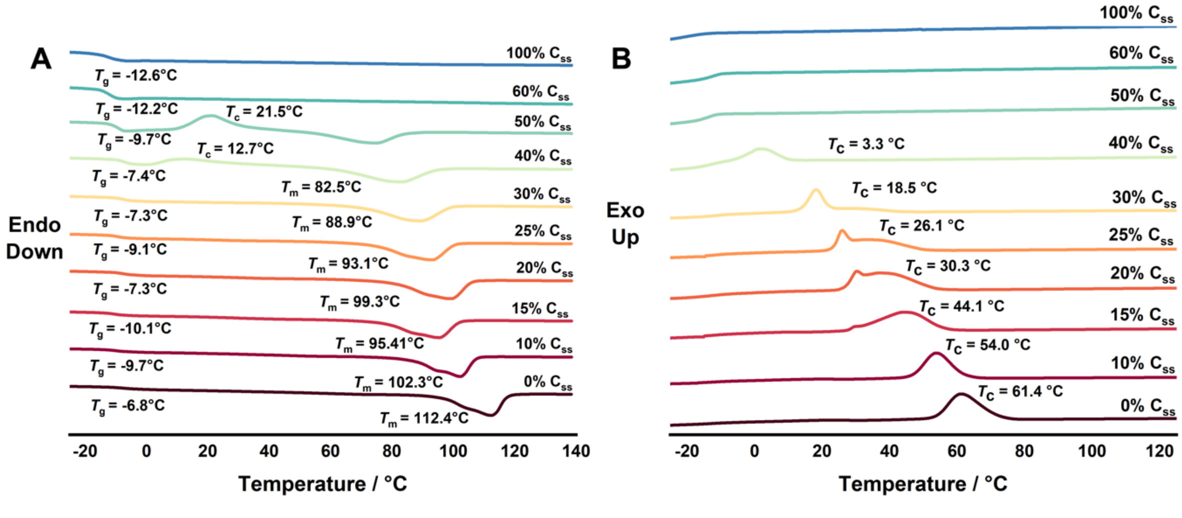


**Supplementary Figure 22.** Differential scanning calorimetry (DSC) thermograms of all synthesized polymers to determine glass transition, melting, and crystalline temperature for each species by 2 cycles of heating. Shown is the second heating cycle (A) and first cooling cycle (B).

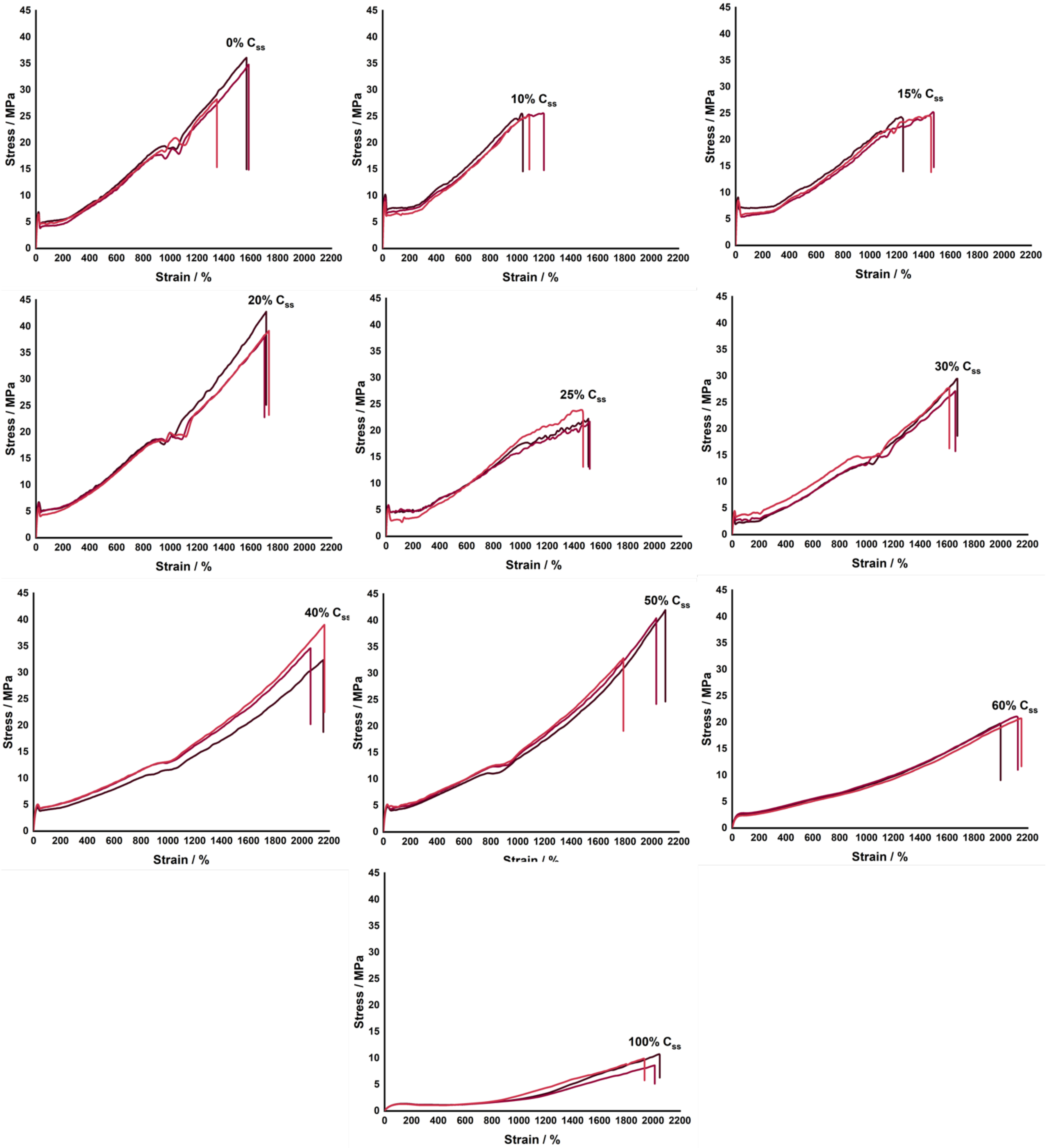


**Supplementary Figure 23.** Stress vs. strain curves for (co)polymers for all synthesized copolymers tested at 10 mm/min. Data for 3 samples are shown to illustrate the reproducibility.

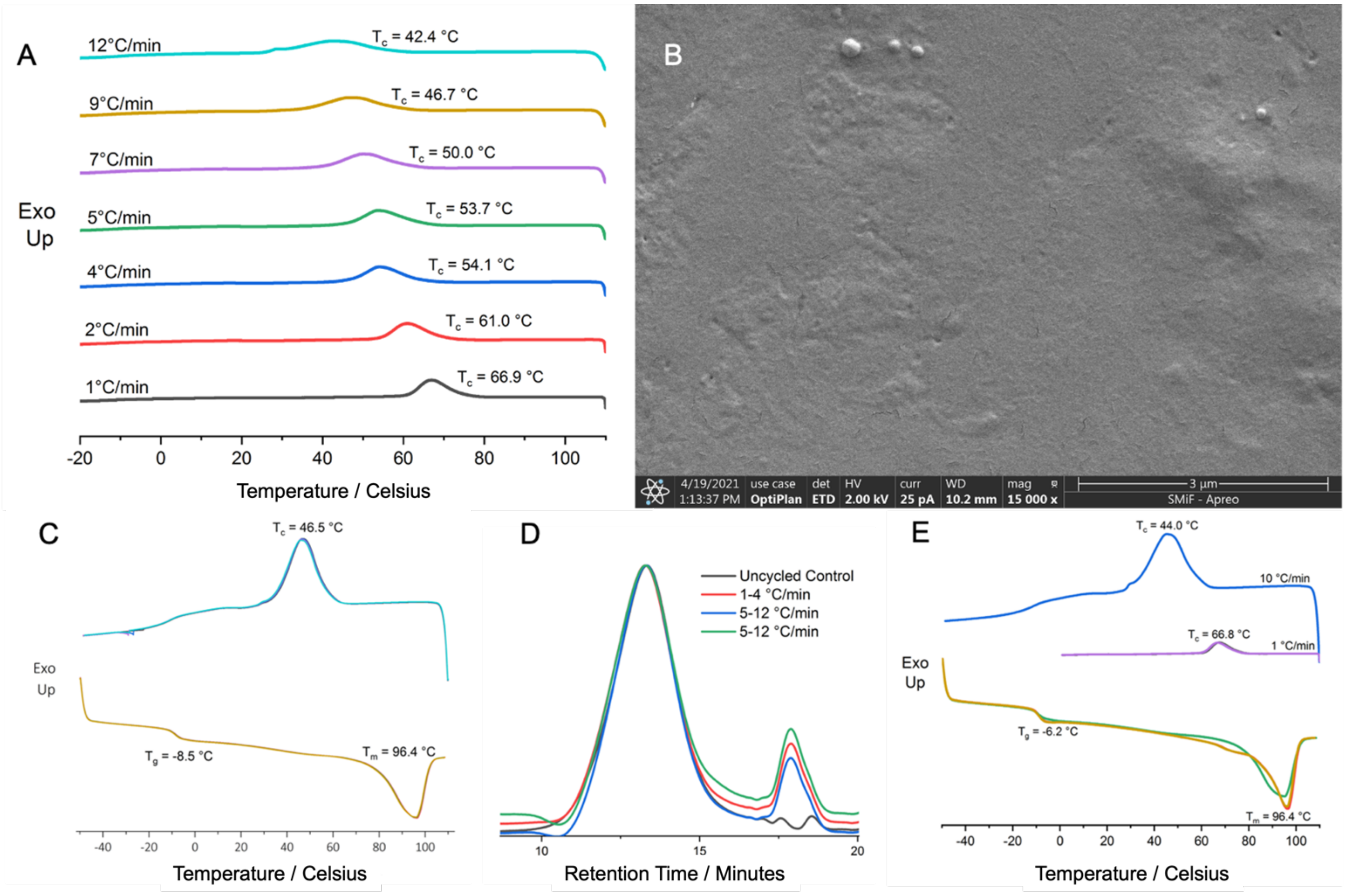


**Supplementary Figure 24.** Investigation into the effect of cooling rate on crystallization of 20% **$C_{ss}$.** **A.** Cooling DSC thermograms from the variable cooling rate DSC experiments (Figure 3 in main text) **B.** SEM image of DSC cooled 20% **$C_{ss}$** **C.** DSC thermogram of 20% **$C_{ss}$** cycled four times to confirm stability throughout cycling **D.** SEC chromatogram of uncycled and cycled 20% **$C_{ss}$** to confirm minimal molecular weight loss after cycling **E.** DSC thermogram to confirm the reproducibility of the $T_c$ shift

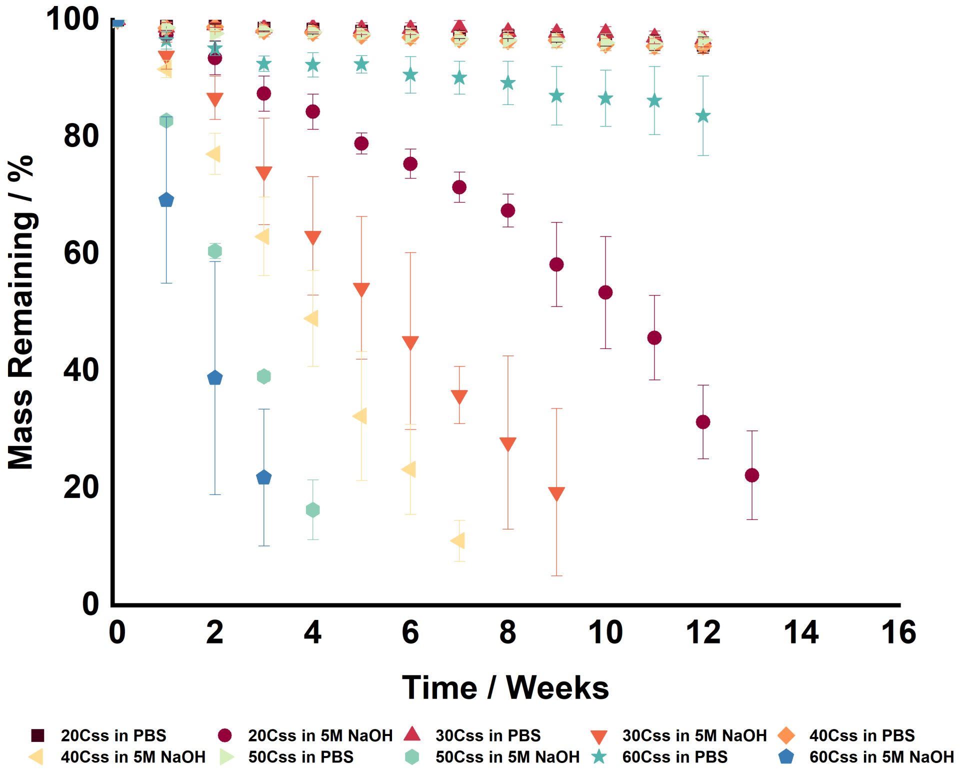


**Supplementary Figure 25.** *In vitro* degradation studies under accelerated (5M NaOH) and physiological conditions (PBS) for a subset of $C_{ss}$ copolymers. Error bars represent the standard deviation of three replicates.

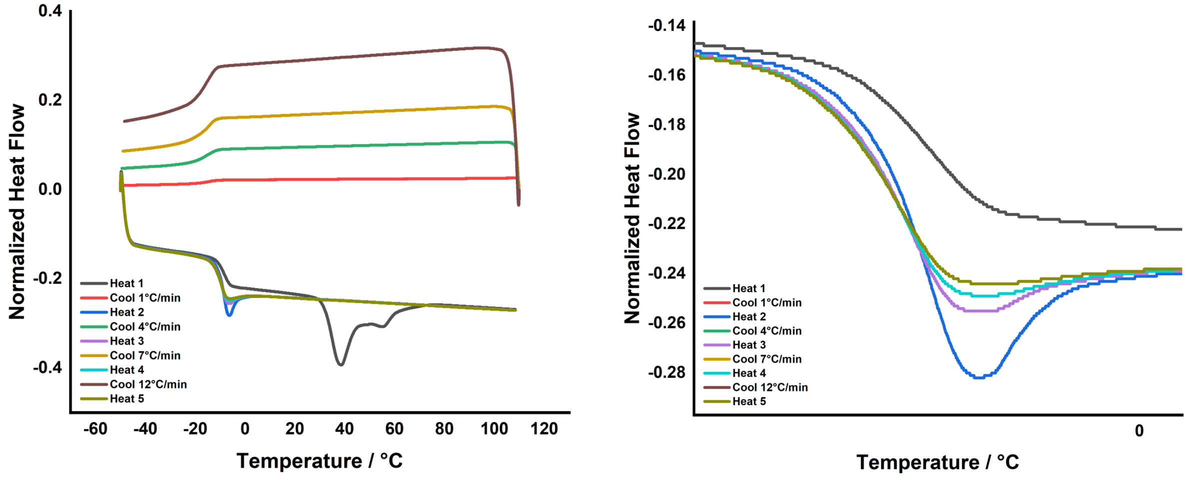


**Supplementary Figure 26.** Variable cooling rate DSC experiment of 60% $C_{ss}$ showing that a “melt”-like transition at the $T_g$ dependent on cooling rate and could be attributed to molecular relaxation of the polymer from stresses/order introduced to it through slow cooling.[1]

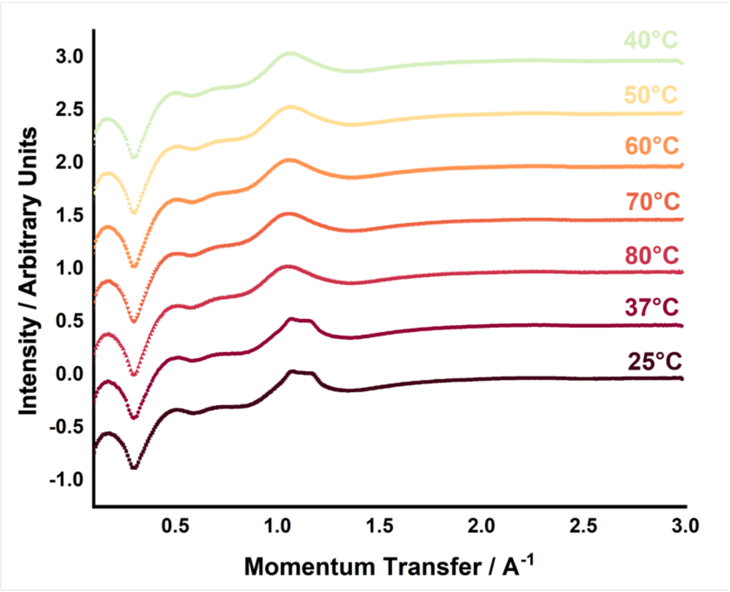


**Supplementary Figure 27.** VT-WAXS experiments of 60% $C_{ss}$ showing changes in the short-range order as a result of heating

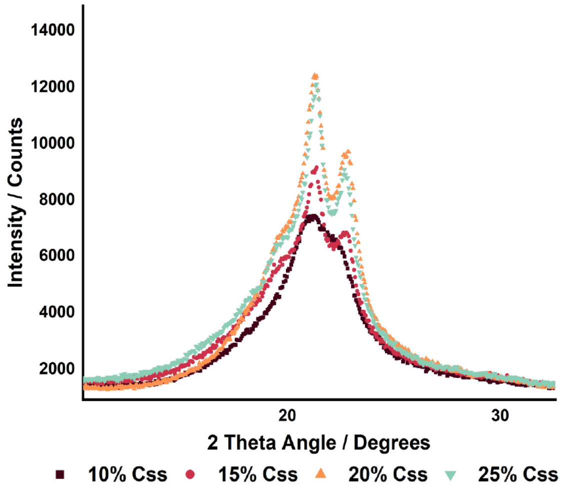


**Supplementary Figure 28.** X-ray diffraction of 10-25% $C_{ss}$ showing that 20% $C_{ss}$ is the most crystalline copolymer.

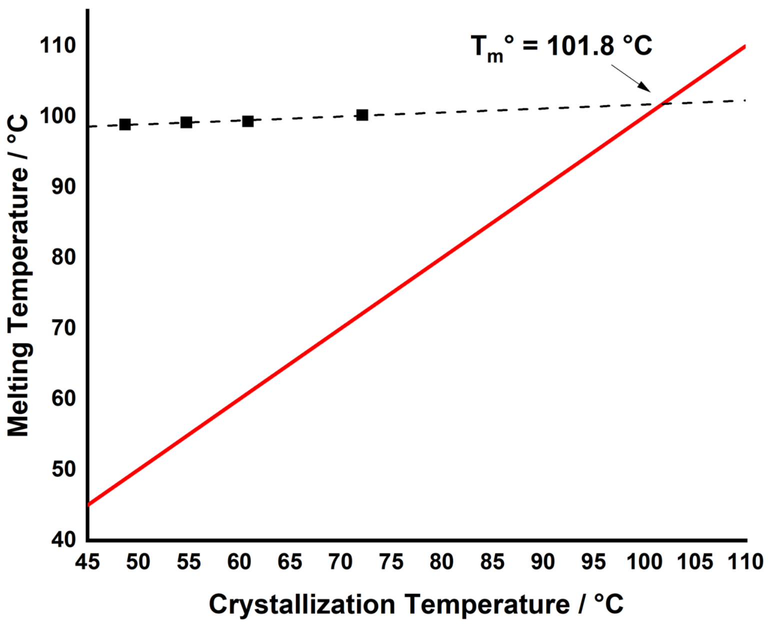


**Supplementary Figure 29.** Representative Hoffman-Weeks extrapolation for $T_m^\circ$ shown in Table 2

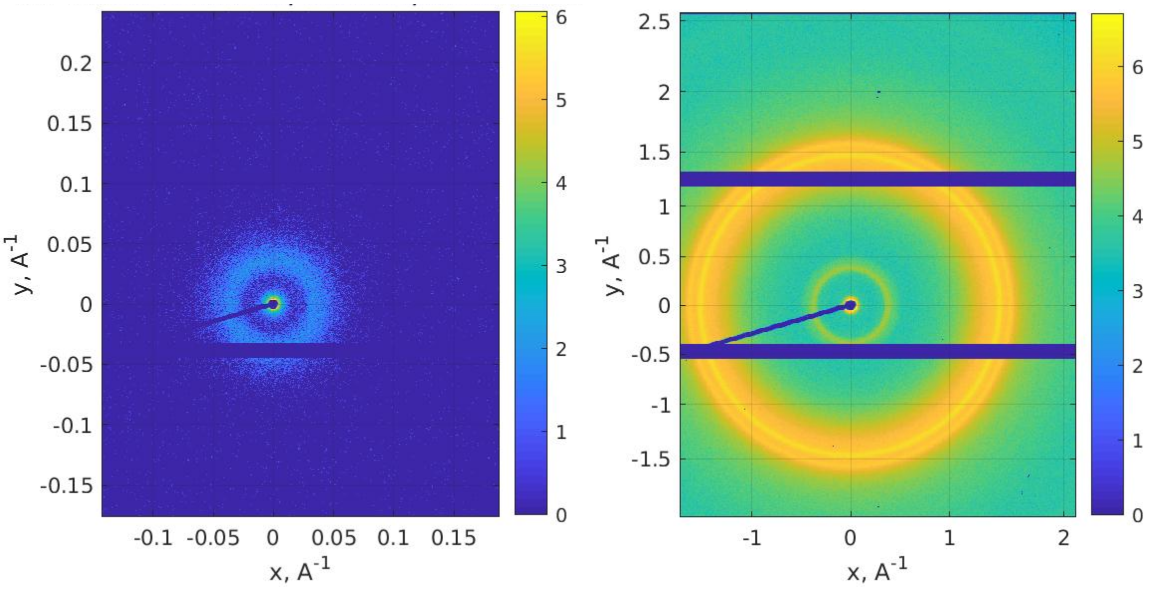


**Supplementary Figure 30.** 2D SAXS (left) and WAXS (right) Scattering Patterns of 20% $C_{ss}$ copolymer

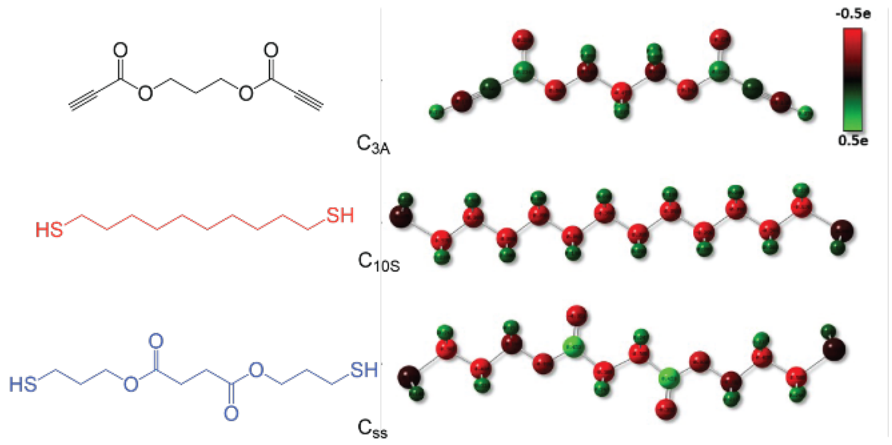


**Supplementary Figure 31. Partial charge distribution for selected monomer sequences**. Magnitudes of the partial charges are shown by color coding (from red to green).

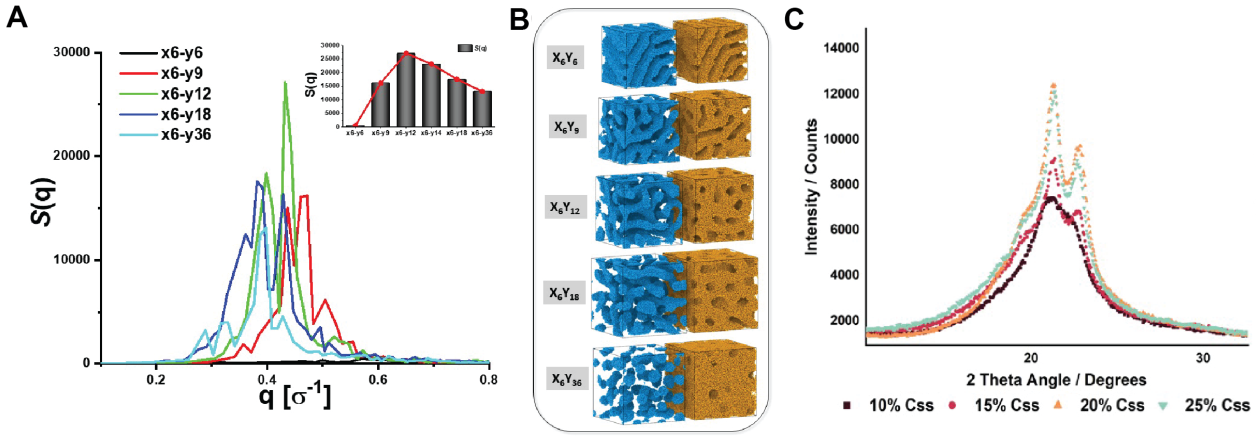


**Supplementary Figure 32. Self-assembly in block copolymer systems:** A. Scattering function S(q) for five self-assembled structures (lamellae, perforated lamellae, gyroid, cylinder, sphere). Inset shows the largest peak height of S(q). Largest peak height corresponds to the gyroid structure. B. Equilibrium structures of the block copolymers with different compositions. (A and B microphases are shown separately for clarity). C. X-ray diffraction of 10 – 25% $C_{ss}$ showing that the 20% $C_{ss}$ copolymer exhibits the greatest amount of crystallinity.

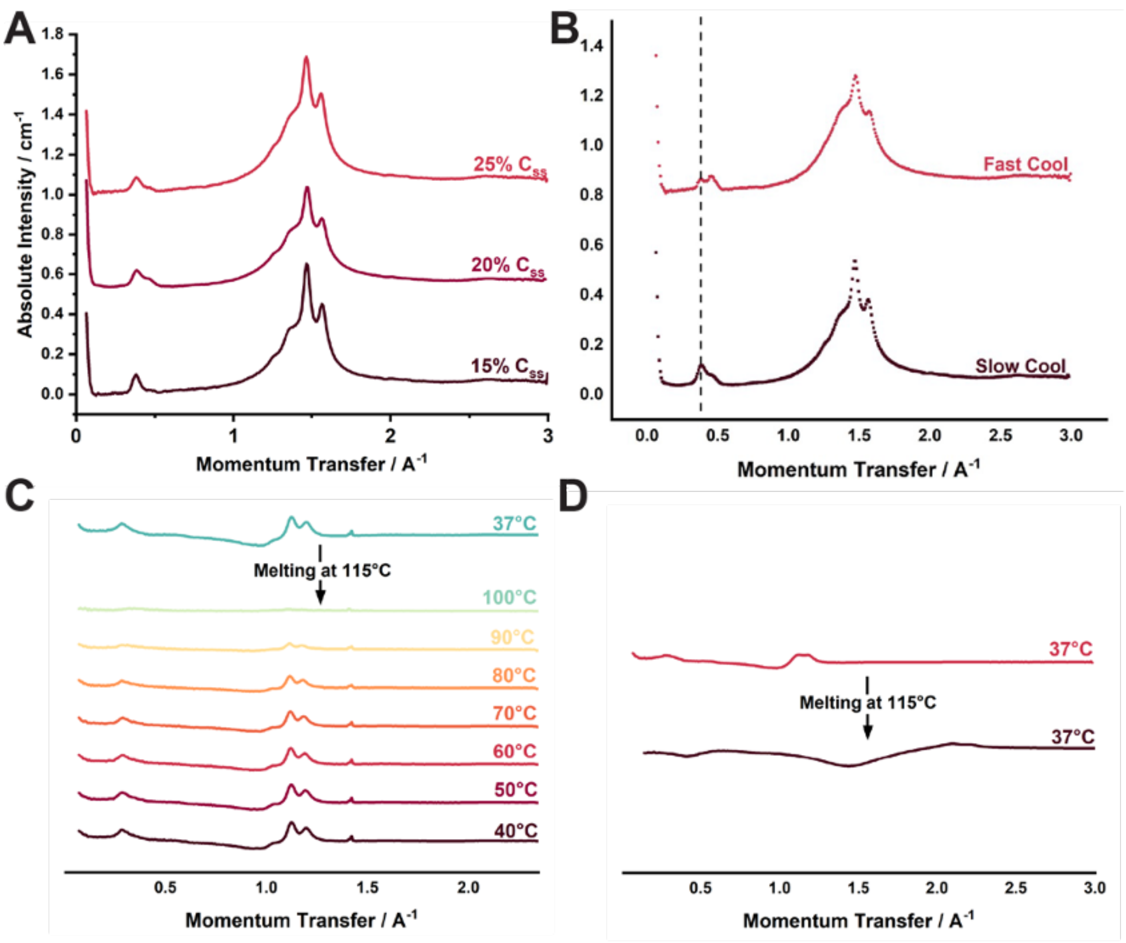


**Supplementary Figure 33. Wide Angle X-Ray Scattering (WAXS) and Compiled Crystalline Properties of 15-25% $C_{ss}$ Copolymers. A**. WAXS of slow cooled 15-25% $C_{ss}$. **B**. WAXS of 20% $C_{ss}$ self-nucleated by DSC, and melt-pressed with slow and fast cooling showing larger dimensional feature for both controlled cooling methods. **C**. VT-WAXS pattern of 20% $C_{ss}$ showing crystal growth via stepped (slow) cooling where the temperatures indicate the temperature at which the scan was taken. **D**. VT-WAXS pattern of 20% $C_{ss}$ showing crystal growth via fast cooling where the temperatures indicate the temperature at which the scan was taken. Greater crystallinity is observed in the slow cooled sample.

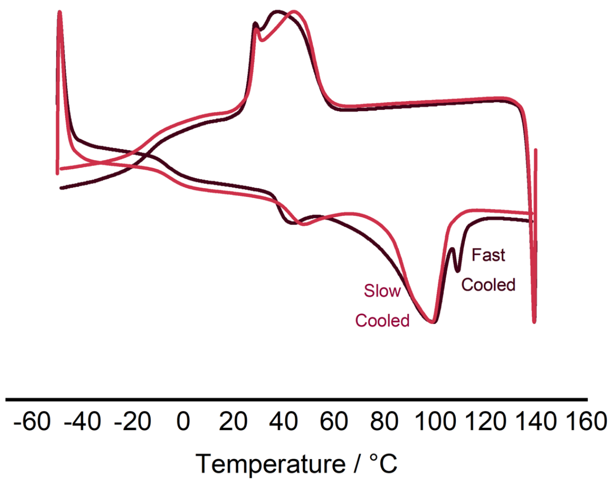


**Supplementary Figure 34. DSC Thermograms for the Slow-Cooled and Fast-Cooled Melt-Pressed 20% $C_{ss}$ Copolymers** The first-heating cycle was used to observe the thermal properties imparted from the melt-processing method used to prepare the tested films. The slow-cooled sample exhibits one polymorph as indicated by the single distribution observed as the $T_m$ curve. The fast-cooled sample shows one dominant polymorph but two overall as demonstrated by the bimodal $T_m$ distribution.

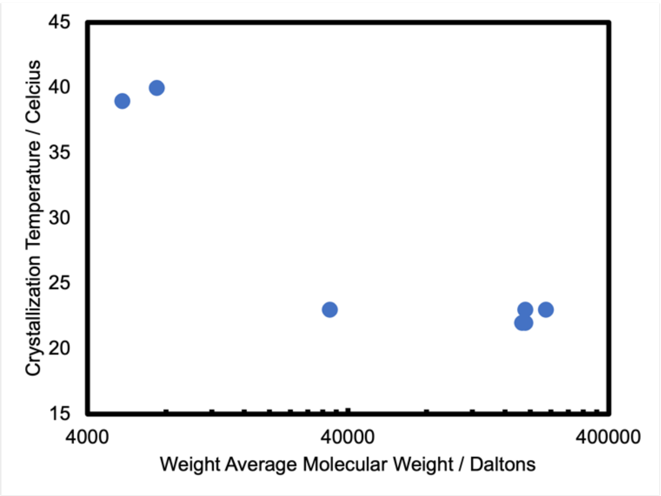


**Supplementary Figure 35. Molecular Weight Study of 20% $C_{ss}$ Copolymers** The effect of molecular weight on crystallization temperature appears to be negligible after ~34 kDa.

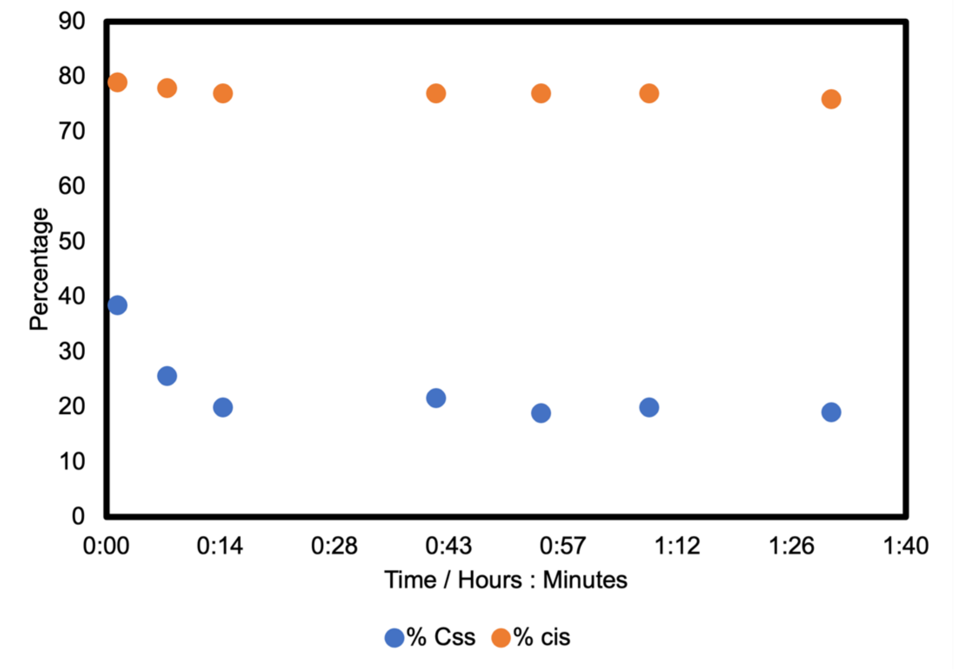


**Supplementary Figure 36. Stoichiometry and alkene conformation over time in 20% $C_{ss}$ Polymerization** There is a slight increased incorporation of the succinate monomer at the beginning of the reaction which plateaus around the expected stoichiometry at the 14-minute mark.

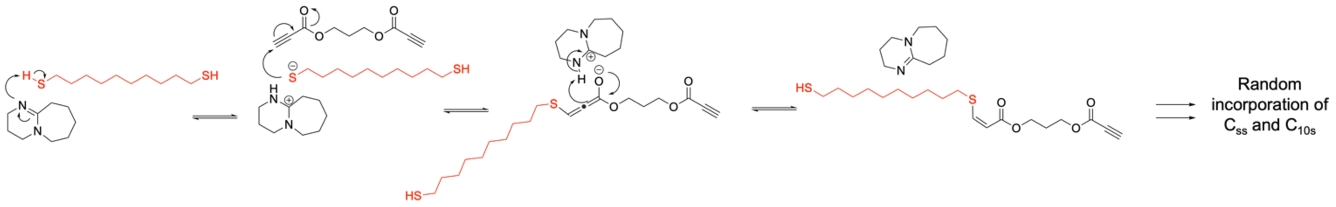


**Supplementary Figure 37.** Proposed mechanism as suggested previously which accounts for the high *cis* content observed for the DBU-catalyzed thiol-yne click polymerizations.[2-4] Shown is one addition cycle which would be repeated many times randomly between $\mathbf{C_{3A}}$ species with either $\mathbf{C_{10s}}$ or $\mathbf{C_{ss}}$.

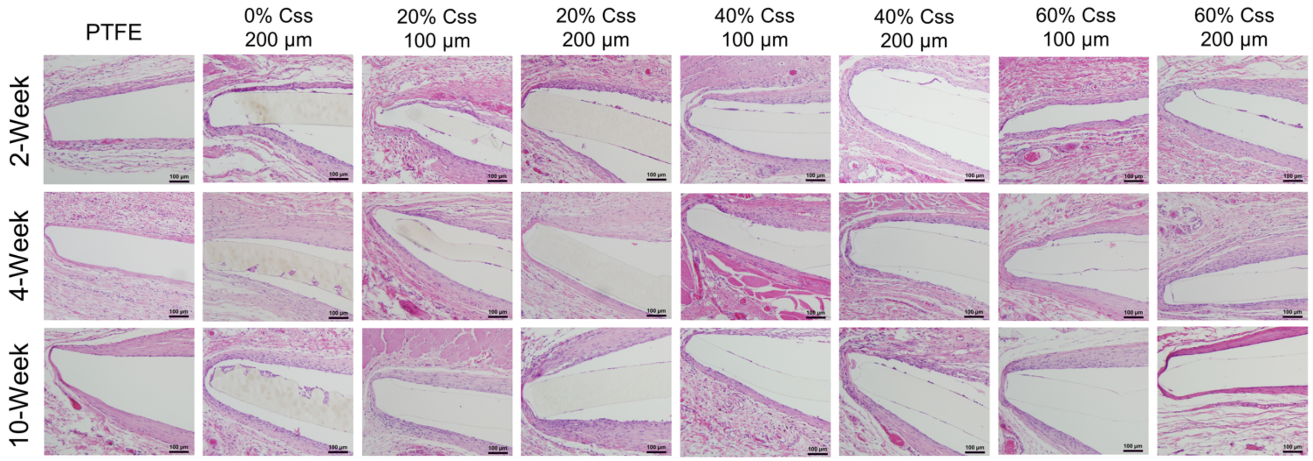


**Supplementary Figure 38. Histopathology of a subset of 100 µm thick $C_{ss}$ copolymer films in comparision to 200 µm thick films.** $C_{ss}$ copolymer disks were implanted subcutaneously for up to 10 weeks to observe their degradation behavior and tissue inflammatory responses *in vivo*. Hemotoxylin & Eosin staining was used to detect fibrous capsule and presence of inflammatory cells based upon ISO 10993-6 standards as assessor metrics (see Supplementary Table 2).

**Supplementary Table 1. Histology scoring of $C_{ss}$ *in vivo* compatibility study.** The presence of inflammatory cells diminished over time of implantation in all groups between week-2 and week-10 timepoints. Necrosis was absent in all sample groups and multinucleated giant cells were either sparse or lacking along with no evidence of immunogenicity. Comparisons to PTFE controls indicated that good biocompatibility and biostability (n=4).

| Polymer | PTFE | | | 0%Css | | | 20%Css | | | 20%Css | | | 40%Css | | | 40%Css | | | 60%Css | | | 60%Css | | |
|---|---|---|---|---|---|---|---|---|---|---|---|---|---|---|---|---|---|---|---|---|---|---|---|---|
| Thickness | | | | 200µm | | | 100µm | | | 200µm | | | 100µm | | | 200µm | | | 100µm | | | 200µm | | |
| Week | 2 | 4 | 10 | 2 | 4 | 10 | 2 | 4 | 10 | 2 | 4 | 10 | 2 | 4 | 10 | 2 | 4 | 10 | 2 | 4 | 10 | 2 | 4 | 10 |
| Lymphocytes | 1 | 1.5 | 1.25 | 1 | 2 | 1.25 | 2 | 1.75 | 1 | 1.75 | 1.75 | 1.5 | 2.25 | 2 | 1.75 | 1.75 | 1.75 | 1.25 | 2 | 1.75 | 1.25 | 1.75 | 2.25 | 1.25 |
| Neutrophils | 1.5 | 0.5 | 1 | 2 | 1 | 1 | 2 | 1.5 | 1.5 | 1.75 | 1.25 | 1 | 1.75 | 1,5 | 0.75 | 1.5 | 1.25 | 0.25 | 2 | 1 | 0.25 | 1.5 | 1.75 | 0.5 |
| Macrophages | 1.75 | 1.25 | 1 | 2.75 | 2 | 2 | 2.5 | 2.25 | 2 | 2.5 | 2 | 1.75 | 2.75 | 2.25 | 2 | 2.25 | 2.25 | 2 | 2.5 | 2.5 | 1.5 | 2.75 | 2.25 | 1.25 |
| Giant Cell | 0 | 0 | 0 | 1.75 | 1.25 | 0.75 | 0.75 | 0 | 0 | 0.5 | 0 | 0 | 1 | 0 | 0 | 0.25 | 0.25 | 0.25 | 0.75 | 0 | 0 | 0.5 | 0 | 0 |

**Supplementary Table 2. Overview of the equilibrium states of the copolymer melt system.**

| x | y | $N_{total}$ | $L$ [σ] | Phase Structure (phase A) |
|---|---|---|---|---|
| 6 | 6 | 153,600 | 56.2 | Lamella |
| 6 | 9 | 192,000 | 61.0 | Perforated Lamella |
| 6 | 12 | 230,400 | 65.0 | Gyroid |
| 6 | 18 | 307,200 | 71.7 | Thread/Cylinder |
| 6 | 36 | 268,800 | 68.8 | Spherical/Island |

## Supplementary Methods

**Spin Coating** Spin-coating was performed on a Laurell Technologies Corporation HL650Mz-23NPPB spin coater with two 5-second 500 rpm at 500 rpm/s cycles followed by one 3-second 2000 rpm at 1500 rpm/s cycle and finally one 30-second 5000 rpm at 2000 rpm/s cycle. The lower speed cycles were performed to reduce the wobble of the spinning substrate when accelerating.

**Degradation study of $C_{ss}$ Copolymers** The selected copolymers were melt-pressed into xx thick films. 8 mm discs were punched out, weighed and submerged in either 1x PBS pH 7.4 or 5 M NaOH solutions. The samples were incubated for preset time points at 37 °C with shaking then rinsed, dried under high vacuum and weighed to track mass loss over time as the polymer degrades.

## SUPPLEMENTARY REFERENCES

(1) *Interpreting Unexpected Events and Transitions in DSC Results*; TA039; Instruments, T.

(2) Worch, J. C.; Stubbs, C. J.; Price, M. J.; Dove, A. P. Click Nucleophilic Conjugate Additions to Activated Alkynes: Exploring Thiol-yne, Amino-yne, and Hydroxyl-yne Reactions from (Bio)Organic to Polymer Chemistry. *Chem Rev* **2021**, *121* (12), 6744-6776. DOI: 10.1021/acs.chemrev.0c01076.

(3) Daglar, O.; Gunay, U. S.; Hizal, G.; Tunca, U.; Durmaz, H. Extremely Rapid Polythioether Synthesis in the Presence of TBD. *Macromolecules* **2019**, *52* (9), 3558-3572. DOI: 10.1021/acs.macromol.9b00293.

(4) Truong, V. X.; Dove, A. P. Organocatalytic, regioselective nucleophilic "click" addition of thiols to propiolic acid esters for polymer-polymer coupling. *Angew Chem Int Ed Engl* **2013**, *52* (15), 4132-4136. DOI: 10.1002/anie.201209239.
