# Supplementary material for: Resorbable barrier polymers for flexible bioelectronics
Source: Nat Commun. 2023 Nov 11;14:7299. doi: 10.1038/s41467-023-42775-5 (PMC10638316; doi:10.1038/s41467-023-42775-5)
Supplement: Supplementary file 3 — Reporting Summary [file 41467_2023_42775_MOESM3_ESM.pdf]

nature portfolio

Corresponding author(s): Matthew L. Becker

Last updated by author(s): Oct 17, 2023

Reporting Summary

Nature Portfolio wishes to improve the reproducibility of the work that we publish. This form provides structure for consistency and transparency in reporting. For further information on Nature Portfolio policies, see our Editorial Policies and the Editorial Policy Checklist.

Statistics

For all statistical analyses, confirm that the following items are present in the figure legend, table legend, main text, or Methods section.

n/a	Confirmed	
☐	☒	The exact sample size (*n*) for each experimental group/condition, given as a discrete number and unit of measurement
☐	☒	A statement on whether measurements were taken from distinct samples or whether the same sample was measured repeatedly
☒	☐	The statistical test(s) used AND whether they are one- or two-sided<br>*Only common tests should be described solely by name; describe more complex techniques in the Methods section.*
☒	☐	A description of all covariates tested
☒	☐	A description of any assumptions or corrections, such as tests of normality and adjustment for multiple comparisons
☐	☒	A full description of the statistical parameters including central tendency (e.g. means) or other basic estimates (e.g. regression coefficient) AND variation (e.g. standard deviation) or associated estimates of uncertainty (e.g. confidence intervals)
☒	☐	For null hypothesis testing, the test statistic (e.g. *F*, *t*, *r*) with confidence intervals, effect sizes, degrees of freedom and *P* value noted<br>*Give P values as exact values whenever suitable.*
☒	☐	For Bayesian analysis, information on the choice of priors and Markov chain Monte Carlo settings
☒	☐	For hierarchical and complex designs, identification of the appropriate level for tests and full reporting of outcomes
☒	☐	Estimates of effect sizes (e.g. Cohen's *d*, Pearson's *r*), indicating how they were calculated

Our web collection on statistics for biologists contains articles on many of the points above.

Software and code

Policy information about availability of computer code

Data collection	DSC, TGA, and DVS data was collected using TRIOS Software, WAXS/SAXS data was collected with saxsgui software v.2.23.33, SEC data was collected with TOSOH EcoSec Elite software, NMR spectroscopy data was collected using Bruker Topspin software with iconNMR, tensile testing was collected using bluehill universal materials testing software, Fityk software was used for SAXS/WAXS fitting
Data analysis	DSC, TGA, and DVS data was analyzed using TRIOS Software, SEC data was analyzed with TOSOH EcoSec Elite software, NMR spectroscopy data was analyzed using mastrenova, tensile testing data was analyzed bluehill universal materials testing software, surface roughness was determined from AFM by Gwyddion, Fityk software was used for SAXS/WAXS fitting

For manuscripts utilizing custom algorithms or software that are central to the research but not yet described in published literature, software must be made available to editors and reviewers. We strongly encourage code deposition in a community repository (e.g. GitHub). See the Nature Portfolio guidelines for submitting code & software for further information.

Data

Policy information about availability of data

All manuscripts must include a data availability statement. This statement should provide the following information, where applicable:

- Accession codes, unique identifiers, or web links for publicly available datasets
- A description of any restrictions on data availability
- For clinical datasets or third party data, please ensure that the statement adheres to our policy

Any data included in the manuscript will be provided upon request. We also used Matweb database which we have indicated in Data Availability Section and accessible here: https://www.matweb.com

Research involving human participants, their data, or biological material

Policy information about studies with human participants or human data. See also policy information about sex, gender (identity/presentation), and sexual orientation and race, ethnicity and racism.

Reporting on sex and gender	N/A
Reporting on race, ethnicity, or other socially relevant groupings	N/A
Population characteristics	N/A
Recruitment	N/A
Ethics oversight	N/A

Note that full information on the approval of the study protocol must also be provided in the manuscript.

Field-specific reporting

Please select the one below that is the best fit for your research. If you are not sure, read the appropriate sections before making your selection.

☒ Life sciences ☐ Behavioural & social sciences ☐ Ecological, evolutionary & environmental sciences

For a reference copy of the document with all sections, see nature.com/documents/nr-reporting-summary-flat.pdf

Life sciences study design

All studies must disclose on these points even when the disclosure is negative.

Sample size	8 Sprague Dawley rats <1 year old were used per experimental group. Animals were selected to provide sufficient substrates for mechanical, molecular mass and histological quantification (4 histology, 4 mechanical tests & molecular mass) per test material per time point. Histology numbers were determined by common ISO standards and expectations. Mechanical test numbers are in line with previous measurements.
Data exclusions	No data were excluded from our in vivo experiments.
Replication	The implant surgeries were performed on two different days by two different physicians, both of whom were blinded to the composition of the implant. All attempts at replication were successful. There were no detectable differences between the physicians.
Randomization	In this study, a robust randomization process was implemented to assign animals to different experimental groups while maintaining an equal distribution of key variables, such as sex and implant placement. The randomization was carried out using a computer-generated randomization scheme, ensuring that each animal is allocated to a specific group in an unbiased and unpredictable manner. The randomization was performed by an individual not directly involved in the study, enhancing the integrity of the allocation process. In this case the animals were named after Greek mythology characters.
Blinding	The surgeon was blinded to the specific implants by a 3rd party naming scheme and the individuals involved in assessing parameters such as mechanical stability, histological samples, and molecular analyses will be blinded to the group allocation of each animal.

Reporting for specific materials, systems and methods

We require information from authors about some types of materials, experimental systems and methods used in many studies. Here, indicate whether each material, system or method listed is relevant to your study. If you are not sure if a list item applies to your research, read the appropriate section before selecting a response.

Materials & experimental systems

n/a	Involved in the study
☒	☐ Antibodies
☒	☐ Eukaryotic cell lines
☒	☐ Palaeontology and archaeology
☐	☒ Animals and other organisms
☒	☐ Clinical data
☒	☐ Dual use research of concern
☒	☐ Plants

Methods

n/a	Involved in the study
☒	☐ ChIP-seq
☒	☐ Flow cytometry
☒	☐ MRI-based neuroimaging

Animals and other research organisms

Policy information about studies involving animals; ARRIVE guidelines recommended for reporting animal research, and Sex and Gender in Research

Laboratory animals	Sprague-Dawley Rats. Animals were housed in pairs in line with Duke Guidelines, and maintained at 23 C and 40% humidity with 12/12 light dark cycles in the vivarium.
Wild animals	Study did not involve wild animals
Reporting on sex	Study contained equal numbers of both sexes, 8 rats per experimental group<br><br>Animals were purchased with a determined sex.
Field-collected samples	N/A
Ethics oversight	Protocol #A168-20-8 approved by Duke Institutional Animal Care and Use Committee (IACUC)

Note that full information on the approval of the study protocol must also be provided in the manuscript.

Plants

Seed stocks	*Report on the source of all seed stocks or other plant material used. If applicable, state the seed stock centre and catalogue number. If plant specimens were collected from the field, describe the collection location, date and sampling procedures.*
Novel plant genotypes	*Describe the methods by which all novel plant genotypes were produced. This includes those generated by transgenic approaches, gene editing, chemical/radiation-based mutagenesis and hybridization. For transgenic lines, describe the transformation method, the number of independent lines analyzed and the generation upon which experiments were performed. For gene-edited lines, describe the editor used, the endogenous sequence targeted for editing, the targeting guide RNA sequence (if applicable) and how the editor was applied.*
Authentication	*Describe any authentication procedures for each seed stock used or novel genotype generated. Describe any experiments used to assess the effect of a mutation and, where applicable, how potential secondary effects (e.g. second site T-DNA insertions, mosiacism, off-target gene editing) were examined.*
