# Supplementary material for: Resorbable barrier polymers for flexible bioelectronics
Source: Nat Commun. 2023 Nov 11;14:7299. doi: 10.1038/s41467-023-42775-5 (PMC10638316; doi:10.1038/s41467-023-42775-5)
Supplement: Supplementary file 2 — Peer Review File [file 41467_2023_42775_MOESM2_ESM.pdf]

nature portfolio

Peer Review File

Resorbable Barrier Polymers for Flexible Bioelectronics

Open Access This file is licensed under a Creative Commons Attribution 4.0 International License, which permits use, sharing, adaptation, distribution and reproduction in any medium or format, as long as you give appropriate credit to the original author(s) and the source, provide a link to the Creative Commons license, and indicate if changes were made. In the cases where the authors are anonymous, such as is the case for the reports of anonymous peer reviewers, author attribution should be to 'Anonymous Referee' followed by a clear attribution to the source work. The images or other third party material in this file are included in the article's Creative Commons license, unless indicated otherwise in a credit line to the material. If material is not included in the article's Creative Commons license and your intended use is not permitted by statutory regulation or exceeds the permitted use, you will need to obtain permission directly from the copyright holder. To view a copy of this license, visit http://creativecommons.org/licenses/by/4.0/.

REVIEWER COMMENTS

Reviewer #1 (Remarks to the Author):

Rogers, Becker, and coauthors present a succinate-based resorbable polymer for transient flexible electronics. A family of polymers with varying incorporation of the succinate monomer (Css) and hydrophobic monomer (C10S) was prepared by thiol-yne polymerization. The polymers were systematically characterized, with detailed information included in the SI. The thermal and mechanical properties were evaluated, with insights on morphology where appropriate. Personally, I found the motivation, concept, execution, and application to be exceptional and more than appropriate for publication in Nature Communication. I think this is an important contribution to the field, and brings attention to the need to develop new polymers for these applications. The authors also nicely included all the relevant experiments.

I found the data in Table 1 to be well-organized, but slightly difficult to pull conclusions. This is in large part due to the Mw differences, which may be responsible for the changes in several of these columns. It would be excessive to request a molecular weight study, but can the authors include a few sentences to acknowledge this and comment on how Mw can affect these properties. If a partial Mw study is available from the preparation of this manuscript, it may be useful in the SI.

While succinate is an attractive monomer feedstock because it is a Krebs cycle intermediate, C10S is not. For the device work (Figure 7), the copolymer used has 80% of the C10S monomer, not the Css monomer. Overall, this is suitable, given the biocompatibility studies; but a few sentences of this acknowledgement would be appreciated.

For my files, there are some unreadable icons in the molecular dynamics section. I suggest uploading a PDF version.

For Figure 4B, the relative humidity line may be difficult to correlate to a different axis. I suggest place an arrow.

I found the introduction of the dynamic vapor experiments and the connection between these two paragraphs harder to follow relative the rest of the manuscript. Figure 4B is discussed before 4A. Why are there two lines for each of the data in 4A—can you include additional sentences to describe the data? Can the arrows for the image on 4A point into the actual material? At first glance, I thought the light beige was both the Css copolymer and marine epoxy.

While some of the graphs have markers to help identify the lines (4A), some lines may be difficult to distinguish from a color perspective. Having some of the lines dashed may help with this.

Some additional labels on the figures may be helpful. For example, Figure 2C could be more informational.

Reference 25 is oddly all capitalized. It would be nice to see more diversity in the citations (12 of the references are either from Becker or Rogers group, 1/3 of the references).

I think it would be interesting to add a few sentences comparing this material to PCL-based elastomers/substrates or other materials that are appropriate but yet to be tested for water barrier properties.

Reviewer #2 (Remarks to the Author):

The manuscript describes an interesting approach for the preparation of barrier polymers for bioelectronics applications that can be absorbed once they have fulfilled their function. Although the synthesis method of similar copolyesters has been previously described by the same authors (reference 18), its application for the preparation of flexible films with controlled mechanical, thermal, and degradability properties, as well as its use as pressure sensors, has not been described before. The newly prepared films are biocompatible an exhibit significantly improved durability after implantation compared to the various coatings described in the literature. The manuscript is well written, the results are comprehensively explained and the subject can be considered as a 'hot topic'. I have just some questions that I would like to be answered before the manuscript being accepted in Nature Communications.

(1) In the supplementary material, there is quite interesting information that is not mentioned throughout the manuscript, such as the characterization by 1H NMR or the in vitro hydrolytic degradation studies of the synthesized copolymers. The authors should reference this information.

(2) What is the microstructure of the synthesized copolyesters? Are they random, alternating, or block copolymers? It would be advisable for the authors to analyze some of the synthesized copolymers using 13C NMR. The signals corresponding to the carbonyl group of the C3A unit should exhibit splitting due to sequence distribution, which would allow discerning the microstructure of the synthesized copolymers.

(3) ¿Do the authors have an explanation for the unusual mechanical behavior of the C3A with C10S polyester, which exhibits a lower modulus and higher elongation at break compared to the copolyester containing 10% Css?

(4) The authors should indicate how they determined the decomposition temperature of the synthesized polymers. Is it the temperature of maximum degradation rate? This information should be included in Table 1.

(5) The authors should thoroughly review the captions of Figures 2 and 3 as there seems to be a mismatch between the captions and the indicated figures.

Reviewer #3 (Remarks to the Author):

Major comments:

1. The authors presented the Tg, Tm, Tc, E0, εb of the Css copolymers as a function of Css ratio in Table 1, however, the molecular weight of the polymers varied significantly. Considering that molecular weight generally influences the thermal and mechanical properties of polymers, it would be beneficial if the authors could discuss the impact of molecular weight in the manuscript.

2. Considering the potential applications of these polymers in vivo, the measured elastic moduli (several tens of MPa) are too high, which may lead to a mechanical mismatch with biological tissues (which typically have elastic moduli in the range of several tens of kPa). Please provide an explanation for this issue.

3. Although the crystallinity of the polymers increases with an increasing Css ratio, as demonstrated in Figure 2E, the water contact angles did not exhibit a clear trend (15%, 82o; 20%, 89o; 25%, 63o). Furthermore, those values cannot be referred to as ‘hydrophobic’, since, in general, surfaces with contact angles < 90o are designated as hydrophilic.

4. The authors compared the root mean square (RMS) roughness of the slow-cooled Css polymer with that of polyimide film. However, the roughness value of the polyimide film (~50 um) appears to be abnormally high. To address this, the authors should provide information about the manufacturer, fabrication method, thickness of the polyimide film.

5. In Figure 4A, it seems that the authors conducted the encapsulation test twice for each Css ratio. However, the results exhibited inconsistency, which could not draw a conclusion that the 20% Css copolymer extended the lifetime over 40 days. Considering previous reports on polymer encapsulants (e.g., https://doi.org/10.1002/adfm.202000941) and the results shown in Figures 4A and 7B, it is supposed that the functional lifetime of 20% Css copolymer would be less than 10 days, which substantially contradicts a key aspect of the manuscript. The authors should address this discrepancy.

6. The manuscript lacks a comparative analysis of Css copolymer with other polymers of similar thickness in terms of flexibility, elasticity, and hydrophobicity. Incorporating such analysis would improve the manuscript.

Minor comments:

1. The authors should mention the full names of each abbreviation once in the manuscript (e.g., Xc, B3LYP 6-31G, and AM1).

2. Figures 2A and 2B should be switched.

3. The font size in figures throughout the manuscript should be increased to ensure clear visualization. For example, the legends in Figures 3A and 3B are difficult to read.

4. The style of figure captions lacks consistency. Please revise and standardize the figure captions throughout the manuscript.

5. The figure caption for Figure 3 missed an explanation of the SEM images.

6. The figure caption title for Figure 4 does not represent the Figures 4A and 4B.

7. Figure 4A should be discussed before Figure 4B.

8. In the figure caption for Figure 4A, there is a typo: “37 C”. Please correct it.

9. The authors should specify the type of sensor used for the prototype demonstration.

REVIEWER COMMENTS

Reviewer #1 (Remarks to the Author):

Rogers, Becker, and coauthors present a succinate-based resorbable polymer for transient flexible electronics. A family of polymers with varying incorporation of the succinate monomer (Css) and hydrophobic monomer (C10S) was prepared by thiol-yne polymerization. The polymers were systematically characterized, with detailed information included in the SI. The thermal and mechanical properties were evaluated, with insights on morphology where appropriate. Personally, I found the motivation, concept, execution, and application to be exceptional and more than appropriate for publication in Nature Communication. I think this is an important contribution to the field, and brings attention to the need to develop new polymers for these applications. The authors also nicely included all the relevant experiments.

I found the data in Table 1 to be well-organized, but slightly difficult to pull conclusions. This is in large part due to the Mw differences, which may be responsible for the changes in several of these columns. It would be excessive to request a molecular weight study, but can the authors include a few sentences to acknowledge this and comment on how Mw can affect these properties. If a partial Mw study is available from the preparation of this manuscript, it may be useful in the SI.

*Thank you for this point of feedback! We agree that differences in molecular weight (57 – 94 kDa, most around 68-80 kDa) could affect some of the results presented in **Table 1**. The synthesized copolymers provide a clear trend in thermal properties despite differences in molecular weight. This was supported by a small molecular weight study (Figure S35) which showed that crystallization temperature plateaus after ~34 kDa for the 20% C_{ss} stoichiometry. We believe this lends some insight into the molecular weight of entanglement for this system and thus, the variation in properties as a result might be a smaller effect than variations resulting from compositional differences.*

The tensile properties for this system show a general trend in the way mentioned in-text. However, we agree that the variation in low succinate stoichiometries is puzzling. Given that the thermal properties of the materials (which are run under highly controlled conditions due to the nature of DSC measurements) are so consistent, we are inclined to believe that these differences more likely arise from subtle differences in processing, time between processing and measurement, and cooling rate of the prepared samples. While we cannot definitively say exactly what caused the observed differences, the data we present in this paper suggest that these parameters can have a big effect on polymer properties. It would be very interesting as future work to explore these ramifications more thoroughly.

We have adjusted the language when addressing this trend to reflect that it a general trend.

"Young's moduli (E0) and ultimate tensile strengths generally decreased with increasing Css stoichiometry accompanied by a general increase in elongation at break (ε_{break}). This is expected given the trends observed in crystallinity."

While succinate is an attractive monomer feedstock because it is a Krebs cycle intermediate, C10S is not. For the device work (Figure 7), the copolymer used has 80% of the C10S monomer, not the Css monomer. Overall, this is suitable, given the biocompatibility studies; but a few sentences of this acknowledgement would be appreciated.

We agree! We have created a Challenges section in which we discuss the limitations of this study more explicitly and have included this point in that section.

"This system investigates a narrow monomer scope which balances a metabolizable monomer (C_{ss}) with a non-metabolizable monomer (C_{10s}). The most promising stoichiometry (20% succinate monomer content) contains relatively low amounts of the metabolizable monomer. Thus, structures which increase the amount of resorbable components will be an active area of future work towards increasing tunability in degradation time."

For my files, there are some unreadable icons in the molecular dynamics section. I suggest uploading a PDF version.

For Figure 4B, the relative humidity line may be difficult to correlate to a different axis. I suggest place an arrow.

We have added this to both DVS figures to improve quick understanding of our figure!

I found the introduction of the dynamic vapor experiments and the connection between these two paragraphs harder to follow relative the rest of the manuscript. Figure 4B is discussed before 4A. Why are there two lines for each of the data in 4A—can you include additional sentences to describe the data? Can the arrows for the image on 4A point into the actual material? At first glance, I thought the light beige was both the Css copolymer and marine epoxy.

We agree and struggled with a good transition between the more routine polymer characterization work and the more application-centered topics. We have opted to add subheadings to help with this transition and also aid in readers quickly finding the information they are interested in. We have also made the suggested figure adjustments and changed the order of the figures within Figure 4.

While some of the graphs have markers to help identify the lines (4A), some lines may be difficult to distinguish from a color perspective. Having some of the lines dashed may help with this.

Thank you for this suggestion, we agree that adding labels/markers increases the accessibility of our figures and have done so for both sets of DVS figures.

Some additional labels on the figures may be helpful. For example, Figure 2C could be more informational.

We have added 'Broad isotropic rings indicate polycrystallinity to Figure 2C'

Reference 25 is oddly all capitalized. It would be nice to see more diversity in the citations (12 of the references are either from Becker or Rogers group, 1/3 of the references).

Thank you for mentioning this. We should have done a better job at comprehensively capturing this field and have added the following references to rectify this.[1-6]

(1) Banjo, A. D.; Agrawal, V.; Auad, M. L.; Celestine, A.-D. N. Moisture-induced changes in the mechanical behavior of 3D printed polymers. *Composites Part C: Open Access* **2022**, *7*. DOI: 10.1016/j.jcomc.2022.100243.
(2) Han, W. B.; Ko, G. J.; Lee, K. G.; Kim, D.; Lee, J. H.; Yang, S. M.; Kim, D. J.; Shin, J. W.; Jang, T. M.; Han, S.; et al. Ultra-stretchable and biodegradable elastomers for soft, transient electronics. *Nat Commun* **2023**, *14* (1), 2263. DOI: 10.1038/s41467-023-38040-4 From NLM Medline.

(3) Han, W. B.; Ko, G. J.; Yang, S. M.; Kang, H.; Lee, J. H.; Shin, J. W.; Jang, T. M.; Han, S.; Kim, D. J.; Lim, J. H.; et al. Micropatterned Elastomeric Composites for Encapsulation of Transient Electronics. *ACS Nano* **2023**, *17* (15), 14822-14830. DOI: 10.1021/acsnano.3c03063
(4) Ponjavic, M.; Nikolic, M. S.; Jeremic, S.; Djokic, L.; Nikodinovic-Runic, J.; Cosovic, V. R.; Djonlagic, J. Influence of Short Central PEO Segment on Hydrolytic and Enzymatic Degradation of Triblock PCL Copolymers. *J. Polym. Environ.* **2017**, *26* (6), 2346-2359. DOI: 10.1007/s10924-017-1130-2.
(5) Shin, J. W.; Chan Choe, J.; Lee, J. H.; Han, W. B.; Jang, T. M.; Ko, G. J.; Yang, S. M.; Kim, Y. G.; Joo, J.; Lim, B. H.; et al. Biologically Safe, Degradable Self-Destruction System for On-Demand, Programmable Transient Electronics. *ACS Nano* **2021**, *15* (12), 19310-19320. DOI: 10.1021/acsnano.1c05463.
(6) Tran, H.; Feig, V. R.; Liu, K.; Wu, H. C.; Chen, R.; Xu, J.; Deisseroth, K.; Bao, Z. Stretchable and Fully Degradable Semiconductors for Transient Electronics. *ACS Cent Sci* **2019**, *5* (11), 1884-1891. DOI: 10.1021/acscentsci.9b00850 From NLM PubMed-not-MEDLINE.

I think it would be interesting to add a few sentences comparing this material to PCL-based elastomers/substrates or other materials that are appropriate but yet to be tested for water barrier properties.

Discussion of this system compared to other systems was added in a new section entitled "Comparison to Other Systems". We have also included an Ashby-type plot which shows where this work falls in comparison to commercially available polymers.

Reviewer #2 (Remarks to the Author):

The manuscript describes an interesting approach for the preparation of barrier polymers for bioelectronics applications that can be absorbed once they have fulfilled their function. Although the synthesis method of similar copolyesters has been previously described by the same authors (reference 18), its application for the preparation of flexible films with controlled mechanical, thermal, and degradability properties, as well as its use as pressure sensors, has not been described before. The newly prepared films are biocompatible an exhibit significantly improved durability after implantation compared to the various coatings described in the literature. The manuscript is well written, the results are comprehensively explained and the subject can be considered as a 'hot topic'. I have just some questions that I would like to be answered before the manuscript being accepted in Nature Communications.

(1) In the supplementary material, there is quite interesting information that is not mentioned throughout the manuscript, such as the characterization by ^{1}H NMR or the in vitro hydrolytic degradation studies of the synthesized copolymers. The authors should reference this information.

Thank you for this feedback. You are absolutely right that this should be referenced in the main text. We have included the following:

Results and Discussion:
"Copolymer structure was confirmed by ^{1}H NMR spectroscopy in supplementary information"

"Whereas, in vitro hydrolytic degradation studies trended with C_{ss} stoichiometry indicating slow, tunable degradation profiles (Figure S25) which is favorable for longer-term implants" Added after discussion of thermal degradation properties.

(2) What is the microstructure of the synthesized copolyesters? Are they random, alternating, or block copolymers? It would be advisable for the authors to analyze some of the synthesized copolymers using ^{13}C NMR. The signals corresponding to the carbonyl group of the C3A unit should exhibit splitting due to sequence distribution, which would allow discerning the microstructure of the synthesized copolymers.

Microstructure is something that would be very interesting to investigate further with this system. We were unsure how to use ^{13}C NMR to obtain the microstructure of these copolymers so we opted to take ^{1}H NMR measurements as the reaction proceeded to obtain the small study included in Figure S36. This may indicate that for most of the reaction the copolymers are indeed statistical except for a small region in the beginning of the reaction where decanedithiol is prone to freezing and increases the proportion slightly of incorporated succinate monomer. Given how the step-growth mechanism proceeds (this region being very low molecular weight species) we believe this effect is likely negligible on overall copolymer performance. However, we have added the following sentence to ensure full transparency about this work:

"Timepoints taken during a 20% C_{ss} polymerization suggest that there is a slight increase in the succinate monomer incorporation towards the beginning of the reaction due to decanedithiol's tendency to freeze (Figure S36). However, due to the nature of typical step-growth reactions, the resulting copolymers are likely still statistical in nature."

(3) ¿Do the authors have an explanation for the unusual mechanical behavior of the C3A with C10S polyester, which exhibits a lower modulus and higher elongation at break compared to the copolyester containing 10% Css?

The tensile properties for this system show a general trend in the way mentioned in-text. However, we agree that the variation in low succinate stoichiometries is puzzling. Given that the thermal properties of the materials (which are run under highly controlled conditions due to the nature of DSC measurements) are so consistent, we are inclined to believe that these differences more likely arise from subtle differences in processing, time between processing and measurement, and cooling rate of the prepared samples. While we cannot definitively say exactly what caused the observed differences, the data we present in this paper suggest that these parameters can have a big effect on polymer properties. It would be very interesting as future work to explore these ramifications more thoroughly.

We have adjusted the language when addressing this trend to reflect that it a general trend.

"Young's moduli (E_0) and ultimate tensile strengths generally decreased with increasing Css stoichiometry accompanied by a general increase in elongation at break (ε_{break}). This is expected given the trends observed in crystallinity."

(4) The authors should indicate how they determined the decomposition temperature of the synthesized polymers. Is it the temperature of maximum degradation rate? This information should be included in **Table 1**.

Thank you for this point of feedback, this information was outlined in our supplementary information section but was also added as a subscript to this table.

(5) The authors should thoroughly review the captions of Figures 2 and 3 as there seems to be a mismatch between the captions and the indicated figures.

Thank you for pointing this out, we have adjusted our captions so they are uniform.

Reviewer #3 (Remarks to the Author):

Major comments:
1. The authors presented the T_g, T_m, T_c, E_0, ε_b of the Css copolymers as a function of Css ratio in **Table 1**, however, the molecular weight of the polymers varied significantly. Considering that molecular weight generally influences the thermal and mechanical properties of polymers, it would be beneficial if the authors could discuss the impact of molecular weight in the manuscript.

Thank you for this point of feedback! We agree that differences in molecular weight (57 – 94 kDa, most around 68-80 kDa) could affect some of the results presented in ***Table 1****. The synthesized copolymers provide a clear trend in thermal properties despite differences in molecular weight. This was supported by a small molecular weight study (S35) which showed that crystallization temperature plateaus after ~34 kDa for the 20% C_{ss} stoichiometry. We believe this lends some insight into the molecular weight of entanglement for this system and thus, the variation in properties as a result might be a smaller effect than variations resulting from compositional differences.*

2. Considering the potential applications of these polymers *in vivo*, the measured elastic moduli (several tens of MPa) are too high, which may lead to a mechanical mismatch with biological tissues (which typically have elastic moduli in the range of several tens of kPa). Please provide an explanation for this issue.

This is a very good point for applications within regenerative medicine. We believe mechanical mismatch considerations are not as relevant since cellular infiltration and tissue regeneration are not goals of this work. Even if there is a small amount of fibrosis or capsule formation, we expect this to remodel after the device degrades. We will be looking closely at this in longer term, device specific studies.

3. Although the crystallinity of the polymers increases with an increasing Css ratio, as demonstrated in Figure 2E, the water contact angles did not exhibit a clear trend (15%, 82°; 20%, 89°; 25%, 63°). Furthermore, those values cannot be referred to as 'hydrophobic', since, in general, surfaces with contact angles < 90° are designated as hydrophilic.

Thank you for pointing this out. We have removed this misleading language from our discussion of this copolymer system.

4. The authors compared the root mean square (RMS) roughness of the slow-cooled Css polymer with that of polyimide film. However, the roughness value of the polyimide film (~50 um) appears to be abnormally high. To address this, the authors should provide information about the manufacturer, fabrication method, thickness of the polyimide film.

Thank you for pointing this out. We believe this was a mistake and cannot characterize the film again due to the instrument being down. We have opted to remove this comparison from the manuscript reporting only the RMS of the slow-cooled films which we are confident is correct.

5. In Figure 4A, it seems that the authors conducted the encapsulation test twice for each Css ratio. However, the results exhibited inconsistency, which could not draw a conclusion that the 20% Css copolymer extended the lifetime over 40 days. Considering previous reports on polymer encapsulants (e.g., https://doi.org/10.1002/adfm.202000941) and the results shown in Figures 4A and 7B, it is supposed that the functional lifetime of 20% Css copolymer would be less than 10 days, which substantially contradicts a key aspect of the manuscript. The authors should address this discrepancy.

We have added a Challenges section in which we discuss the limitations of this study more explicitly than was included before. The performance of this system is controlled by a variety of factors including processing temperature, cooling rate, pressure, etc. which are difficult to control in an academic lab. We believe we have presented some promising proof-of-concept results which would be greatly improved by industry-grade processing, commercial clean room film and sample preparation.

6. The manuscript lacks a comparative analysis of Css copolymer with other polymers of similar thickness in terms of flexibility, elasticity, and hydrophobicity. Incorporating such analysis would improve the manuscript.

Discussion of this system compared to other systems was added in a new section entitled "Comparison to Other Systems". We have also included an Ashby-type plot which shows where this work falls in comparison to commercially available polymers.

Minor comments:
1. The authors should mention the full names of each abbreviation once in the manuscript (e.g., Xc, B3LYP 6-31G, and AM1).
Addressed as requested. B3LYP 6-31G is not an abbreviation and thus was left as is.

2. Figures 2A and 2B should be switched.
Addressed as requested.

3. The font size in figures throughout the manuscript should be increased to ensure clear visualization. For example, the legends in Figures 3A and 3B are difficult to read.
We opted to add labels in the plot to make the figures more accessible

4. The style of figure captions lacks consistency. Please revise and standardize the figure captions throughout the manuscript.
Addressed as requested.

5. The figure caption for Figure 3 missed an explanation of the SEM images.
Addressed as requested.

6. The figure caption title for Figure 4 does not represent the Figures 4A and 4B.
Addressed as requested.

7. Figure 4A should be discussed before Figure 4B.
Addressed as requested.

8. In the figure caption for Figure 4A, there is a typo: "37 C". Please correct it.

Addressed as requested.

9. The authors should specify the type of sensor used for the prototype demonstration.
We have clarified "Preliminary performance of the 20% Css copolymer in a Mg-based prototype device powered by near-field induction (Figure 7A-C) demonstrated the sensitivity of this system..."

REVIEWERS' COMMENTS

Reviewer #1 (Remarks to the Author):

My concerns are addressed and I reiterate that this manuscript should be published in Nature Communications.

Reviewer #2 (Remarks to the Author):

The authors have responded to the reviewer's questions and have corrected the article appropriately. Therefore, I consider that the manuscript can be accepted for publication in Nature Communications.

Reviewer #3 (Remarks to the Author):

I believe that the revisions address the points that were raised in the original review. The paper is now suitable to be accepted in nature communications.
